# An alternative neural basis underlying leptin resistance

**DOI:** 10.1016/j.celrep.2025.115863

**Published:** 2025-06-19

**Authors:** Hongli Li, Cunjin Su, Yuanzhong Xu, Mette Q. Ludwig, Jon Davis, Qingchun Tong

**Affiliations:** 1 Brown Foundation of Molecular Medicine for the Prevention of Human Diseases of McGovern Medical School, University of Texas Health Science Center at Houston, Houston, TX 77030, USA; 2 Digital Science & Innovation, Novo Nordisk A/S, Måløv, Denmark; 3 Novo Nordisk, Boston, MA 02215, USA; 4 MD Anderson Cancer Center & UTHealth Houston Graduate School for Biomedical Sciences, University of Texas Health Science at Houston, Houston, TX 77030, USA; 5 Department of Neurobiology and Anatomy of McGovern Medical School, University of Texas Health Science Center at Houston, Houston, TX 77030, USA; 6 These authors contributed equally; 7 Lead contact

## Abstract

Overconsumption of a palatable Western diet, a condition linked to central leptin resistance, contributes extensively to the current obesity epidemic. In this context, intensive efforts have focused on detailing the molecular mechanisms underlying leptin resistance. Here, we demonstrate that chronic inhibition of hypothalamic arcuate GABAergic neurons (Arc^GABA^) effectively reduced diet-induced obesity (DIO). Interestingly, palatable food exposure increased the activity level of Arc^GABA^ neurons, which do not express the leptin receptor (non-LepR neurons; nonresponsive to leptin). Chronic activation of Arc^GABA^ non-LepR neurons led to massive obesity, which was associated with normal leptin-induced pSTAT3 signaling but phenotypic leptin resistance; i.e., high leptin levels failing to reduce obesity. In contrast, chronic inhibition of Arc^GABA^ non-LepR neurons effectively prevented and reversed DIO, suggesting a potential anti-obesity treatment strategy. These results reveal that obesogenic stimulation of Arc^GABA^ non-LepR neurons, even with intact leptin-pSTAT3 signaling, results in obesity, identifying a novel neural basis underlying leptin resistance.

## In brief

The mechanism of leptin resistance, the major contributor to DIO, remains elusive. Li et al. found that leptin receptor-negative Arc GABAergic neurons (non-LepR neurons) were activated by HFD feeding, that their activation caused leptin-resistant obesity, and that inhibition reduced/reversed DIO, revealing aberrant activation of these neurons as an alternative mechanism underlying leptin resistance.

## Graphical Abstract

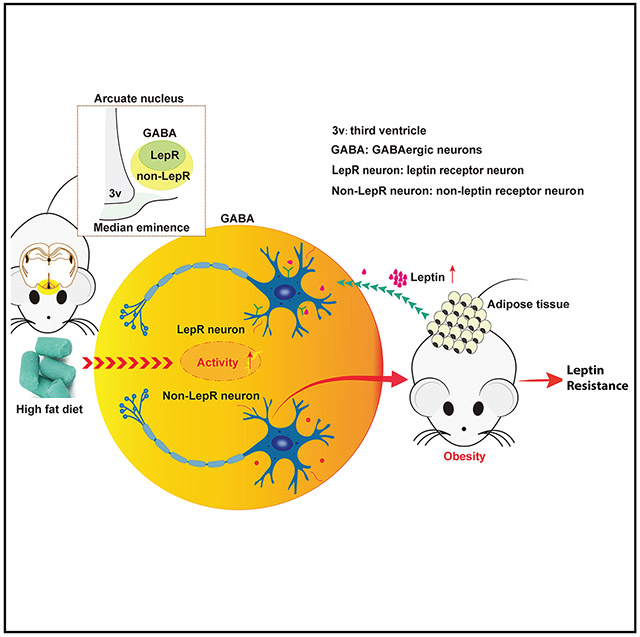

## INTRODUCTION

Body weight homeostasis is controlled by balanced energy intake and expenditure. Obesity occurs when energy intake exceeds expenditure and represents a significant contributor to an array of other metabolic diseases, including type 2 diabetes and cancer.^1^ Despite extensive research on body weight regulation, obesity has reached epidemic proportions, and it is estimated that nearly half of the US adult population will be obese by 2030.^2-4^ In this context, it is well established that excess consumption of palatable Western diets induces an obese phenotype.^5^ In line with this, rodents fed palatable food exhibit similar diet-induced obesity (DIO) with an array of changes in the central nervous system, endocrine signaling mechanisms, and behaviors that mimic clinical obesity.^6^

The discovery of leptin and cloning of the leptin receptor (LepR) provided the first evidence of a neuroendocrine mechanism that regulates body weight.^7-9^ Leptin, secreted mainly from the adipose tissue in proportion to fat mass, is thought to function as a feedback signal on brain neurons that express the LepR (LepR neurons) to reduce food intake, increase energy expenditure, and thereby provide body weight homeostasis.^10^ However, in both human obesity and rodent DIO models, leptin levels are higher than normal, which suggests that leptin, even at a higher level, fails to reduce feeding and/or increase energy expenditure, giving rise to the concept of leptin resistance at the phenotype level.^11-13^ Consistently, the phospho-STAT3 (p-STAT3) signaling pathway, one major leptin-mediated signaling mechanism, is reduced in response to leptin in obese rodents, suggesting the existence of cellular leptin resistance.^14,15^ It is generally accepted that leptin resistance is driven by insufficient activation of signaling cascades, such as p-STAT3 downstream of LepR activation.^14,16,17^ However, despite extensive research, the mechanism underlying leptin resistance remains elusive.

Our previous results demonstrate that chronic activation of arcuate GABAergic (Arc^GABA^) neurons promotes obesity in a redundant manner; i.e., activation of a subset of these neurons is sufficient to induce equivalent body weight gain.^18^ Since it is known that Arc LepR neurons only represent a subset of Arc neurons,^19,20^ we investigated the role of non-LepR neurons in body weight regulation. Our results indicate that palatable food exposure increased the activity levels of Arc non-LepR neurons. Chronic activation of these neurons caused massive obesity, and importantly, the observed obesity is associated with normal p-STAT3-mediated leptin signaling but resistant to leptin treatment, highlighting a dissociation between cellular leptin sensitivity and phenotypic leptin resistance. Interestingly, chronic inhibition of Arc non-LepR neurons prevented body weight gain and reversed obesity in DIO mice. These results demonstrate that dietary stimulation of Arc non-LepR neurons overrides intact leptin’s ability to reduce body weight under obesogenic conditions and highlight this unique neuronal population as a viable therapeutic target for obesity.

## RESULTS

### Chronic inhibition of Arc^GABA^ neurons reduced DIO

Inhibition of Arc^GABA^ neurons reduces body weight in wild-type rodents and normalizes the obesity in leptin-deficient *ob/ob* mice,^18^ suggesting that leptin function may be controlled by this unique set of Arc^GABA^ neurons. To specifically examine the role of these neurons in obesity, we examined chronic inhibition of Arc^GABA^ neurons in development of a DIO phenotype. The expression of Kir2.1 is known to cause effective chronic inhibition of Arc^GABA^ neurons.^21^ Specifically, we injected pAAV-Syn-DIO-Myc/Kir.21*(E224G,Y242F):P2A:dTomato or a control AAV-DIO-GFP virus to the Arc of Vgat-Cre mice and then followed their body weight on a high-fat diet (HFD) (Figure S1A). We found that the Kir2.1-injected mice displayed attenuated body weight gain relative to controls, suggesting that chronic inhibition of Arc^GABA^ neurons is able to prevent development of obesity (Figure S1B). We next examined the effect of Arc^GABA^ inhibition in a pre-existing obese state, using mice maintained on HFD feeding for 8 weeks prior to viral delivery (Figure S1C). Kir2.1-injected mice exhibited a rapid reduction in body weight, which was maintained during the 8-week manipulation period (Figure S1D), These results suggest that chronic inhibition of Arc^GABA^ neurons can be used to both prevent and reduce obesity.

### HFD feeding induced activation of Arc non-LepR neurons

**Figure 1.**
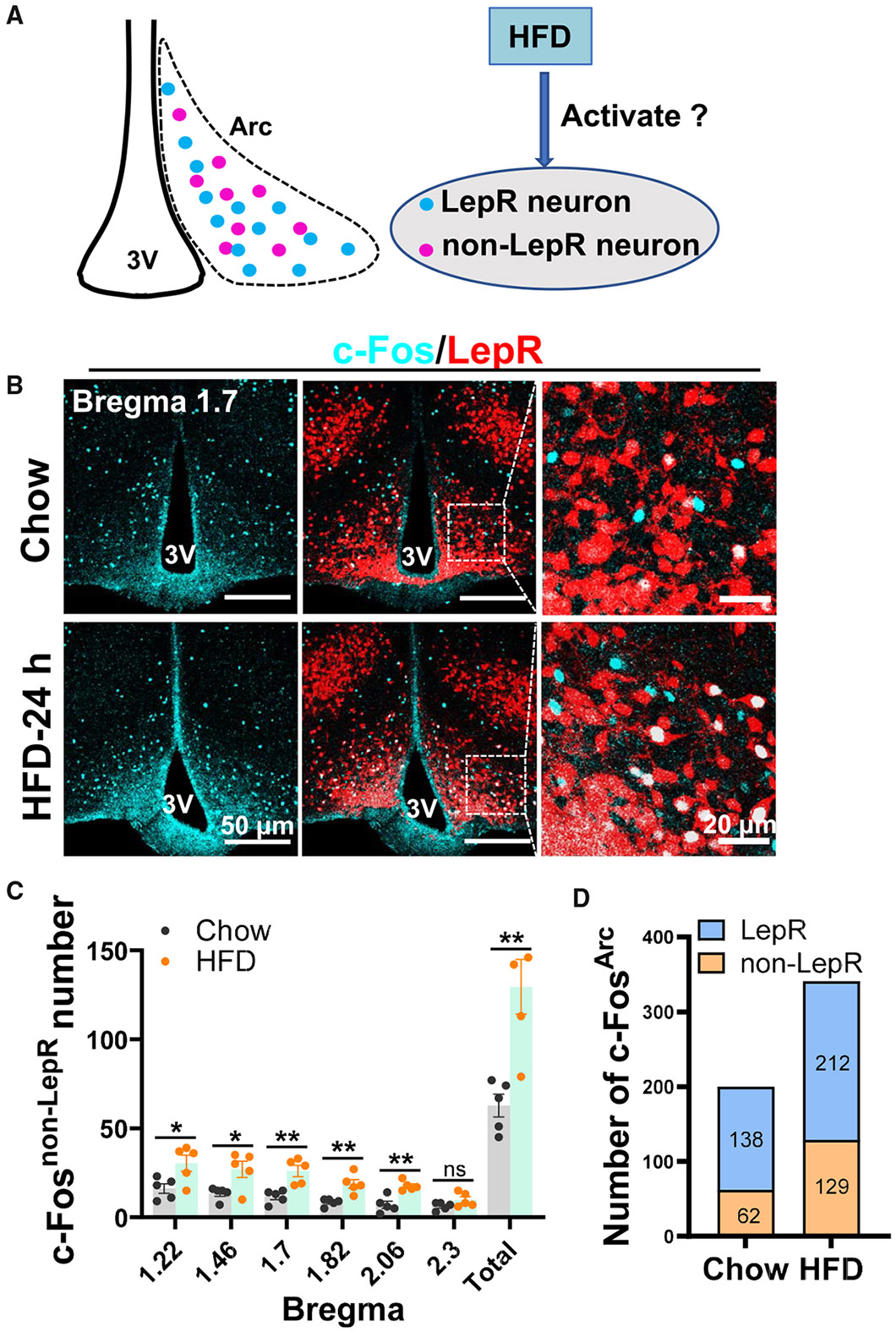
HFD feeding-induced c-Fos expression in Arc non-LepR neurons (A) Diagram depicting the study scheme to examine the effect of HFD feeding on c-Fos expression in Arc LepR and non-LepR neurons. (B) Representative pictures showing c-Fos (blue) and LepR neurons (red) in Arc sections from mice fed chow (top) or 24 h of an HFD (bottom). (C) Statistical comparison of the number of c-Fos-positive neurons at the indicated bregma levels in the Arc between the two groups (*n* = 5 mice/each, unpaired t tests, chow vs. HFD, t (t value) = 2.688, df (degree of freedom) = 8, *p = 0.0276 for bregma 1.22; t = 2.820, df = 8, *p = 0.0225 for bregma 1.46; t = 4.079, df = 8, **p = 0.0035 for bregma 1.7; t = 3.988, df = 8, **p = 0.004 for bregma 1.82; t = 4.536, df = 8, **p = 0.0019 for bregma 2.06; t = 1.782, df = 8; ns (not significant), *p* > 0.05 for bregma 2.3; t = 4.016, df = 8, **p = 0.0039 for total). (D) Overall average number of neurons with c-Fos expression in chow- and HFD-fed mice. 3V, third ventricle; Arc, arcuate nucleus. Scale bars: 50 μM (B, middle and left) or 20 μM (B, right). All data are presented as mean ± SEM (stardard error of the mean).

Previous data suggest that aberrant activation of Arc^GABA^ neurons causes obesity.^18^ Notably, loss of leptin action, as in both *ob/ob* and *db/db* mice, is associated with increased activity of Arc neurons,^22^ suggesting a general role of Arc^GABA^ neuron activation in obesity pathogenesis. Toward this, we examined whether HFD feeding induces activation of Arc neurons. To identify LepR vs. non-LepR neurons, we used LepR-Ires-Cre (LIC)::Ai9 reporter mice and challenged them with an HFD for 24 h before perfusion for c-Fos analysis (Figure 1A). We analyzed c-Fos-expressing neurons in the Arc and found that HFD induced more c-Fos in HFD-fed mice compared to chow diet-fed mice (Figures 1B and S2A). Specifically, the HFD induced significant c-Fos expression in non-LepR neurons (Figures 1C and 1D) and also induced c-Fos expression in LepR neurons, although this did not reach statistical significance (Figure S2B). These data suggest that Arc non-LepR neurons are especially engaged by HFD feeding.

### Chronic activation of non-LepR neurons causes massive obesity

**Figure 2.**
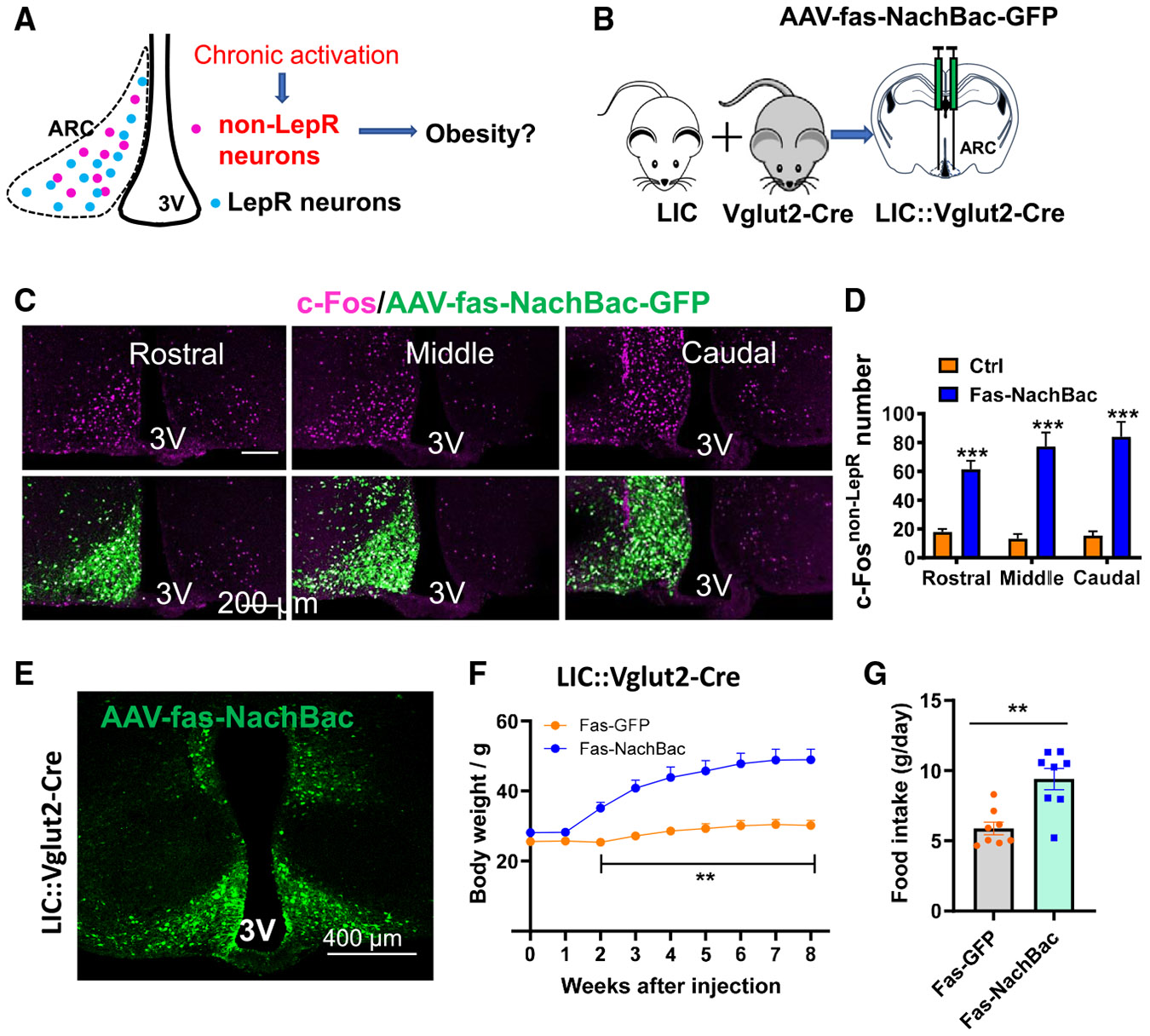
Chronic activation of Arc non-LepR neurons caused massive obesity development (A) Diagram depicting the experimental design to examine the impact on obesity development by selective activation of Arc non-LepR neurons. (B) Diagram showing the strategy to achieve expression of NachBac in Arc non-LepR neurons by delivering the NachBac virus to both sides of the Arc in LIC::Vglut2-Cre mice. (C) Expression pattern of viral expression (GFP) and c-Fos (red), showing c-Fos induction by NachBac expression with viral vector delivery to one side of the Arc in three rostral and caudal sections. Scale bar: 50 μM. (D) Statistical comparison of c-Fos induction between the NachBac-injected side and the control non-injected side (*n* = 5 mice/each, two-way repeated measures (RM) ANOVA, control vs. NachBac, ***p < 0.001). (E) Representative expression patterns of the NachBac virus in the Arc of LIC::Vglut2-Cre mice. Scale bar: 100 μM. (F) Weekly body weight of male LIC::Vglut2-Cre mice with bilateral delivery to the Arc of the NachBac or control virus (two-way RM ANOVA, *n* = 8/each, **p = 0.004 2 weeks post viral injection, ***p < 0001, control vs. NachBac 3–8 weeks post viral injection). (G) Comparison between the groups of mice in feeding (*n* = 8/each, unpaired Student’s t tests, t = 3.990, df = 14, **p = 0.0013). All data are presented as mean ± SEM.

To examine the functional relevance of Arc non-LepR neuron activation to body weight regulation, we generated an animal model allowing for chronic activation of Arc non-LepR neurons (Figure 2A). To achieve this, we generated pAAV-Syn-Fas-Myc/NachBac:P2A:EGFP vectors, which express NachBac and GFP in non-Cre neurons. To prevent off-targeting nearby neurons outside of the Arc, we generated LIC::Vglut2-Cre mice. Since the ventromedial hypothalamus surrounds the Arc and mostly consists of glutamatergic neurons with Vglut2 expression,^23^ the presence of Vglut2-Cre will limit NachBac expression in GABAergic neurons within the Arc (Figure 2B). Indeed, when the NachBac vector was delivered unilaterally to the Arc, the expression was contained within this region and associated with increased c-Fos expression in the injection side (Figure 2C). The increase in c-Fos expression was significant across all bregma levels of the Arc compared to the control side (Figure 2D), consistent with the known action of NachBac in driving chronic neuron activation.^24^

To examine the impact on body weight, we delivered the NachBac virus bilaterally within the Arc (Figures 2E and S3A). Interestingly, we observed that the NachBac-injected mice, when fed chow, developed rapid body weight gain, reaching 50 g 8 weeks after virus delivery, compared to a body weight of approximately 25 g during the same period (Figures 2F, S3B, and S3C). The increased body weight was driven by increased fat mass (Figures S3D and S3E), suggesting development of an obese phenotype. To examine the mechanism of action for the obesity development, we subjected the mice to metabolic measurements using the comprehensive lab animal monitoring system (CLAMS) at a time before statistically significant body weight separation between the two groups (Figures S3F), NachBac-injected mice exhibited increased food intake (Figure 2G) and reduced energy expenditure (Figures S3G and S3H) and locomotion (Figures S3I and S3J). Previous studies suggest that activation of unilateral Arc^GABA^ neurons is sufficient to cause obesity.^18^ To investigate this parameter in Arc non-LepR neurons, we delivered the NachBac virus unilaterally to the Arc of LIC::Vglut2-Cre mice (Figure S4A). In both males (Figures S4B-S4E) and females (Figures S4F and S4G), NachBac injection significantly increased body weight relative to controls (Figures S4B and S4F), an effect associated with increased feeding (Figures S4C and S4G), and increased fat mass (Figure S4D) but no changes in lean mass (Figure S4E). Collectively, these data demonstrate that chronic activation of Arc non-LepR neurons results in massive obesity.

To prevent the effect of potential non-Vglut2-, non-Vgat-positive neurons that were also targeted by fas-NachBac (Figure 2), we generated and delivered pAAV-FlpOn/CreOff-NachBac-EGFP viruses to the Arc of Vgat-Flp::LIC mice to achieve specific targeting of Arc^GABA^ non-LepR neurons with NachBac expression. Specific NachBac expression was confirmed (Figure S5A). The NachBac mice also developed massive obesity (Figure S5B). When measured in TSE Phenomaster cages before body weight divergence (Figure S5C), the NachBac mice exhibited reduced locomotion, especially during the light period (Figures S5D-S5F). Importantly, the NachBac mice showed hyperphagia (Figure S5G) and reduced O_2_ consumption, especially during the dark period (Figures S5H-S5J). These results confirm that Arc^GABA^ non-LepR neurons contribute to the observed obesity phenotype.

To examine the effect of activation of Arc^GABA^ non-LepR neurons on feeding, we delivered pAAV-FlpOn/CreOff-ChR2-EGFP viral vectors to the Arc of Vgat-Flp::LIC mice, which allows specific expression of ChR2-EGFP in Arc^GABA^ non-LepR neurons. Specific viral expression and c-Fos induction by opto-stimulation were confirmed (Figure S6A). Opto-stimulation of Arc^GABA^ non-LepR neurons significantly increased food intake during the 20 min observation period in well-fed animals (Figure S6B) and increased time spent in the food zone (Figure S6C), suggesting that activation of Arc^GABA^ non-LepR neurons is sufficient to promote feeding behavior.

### Obesity induced by activation of non-LepR neurons is associated with normal leptin sensitivity

**Figure 3.**
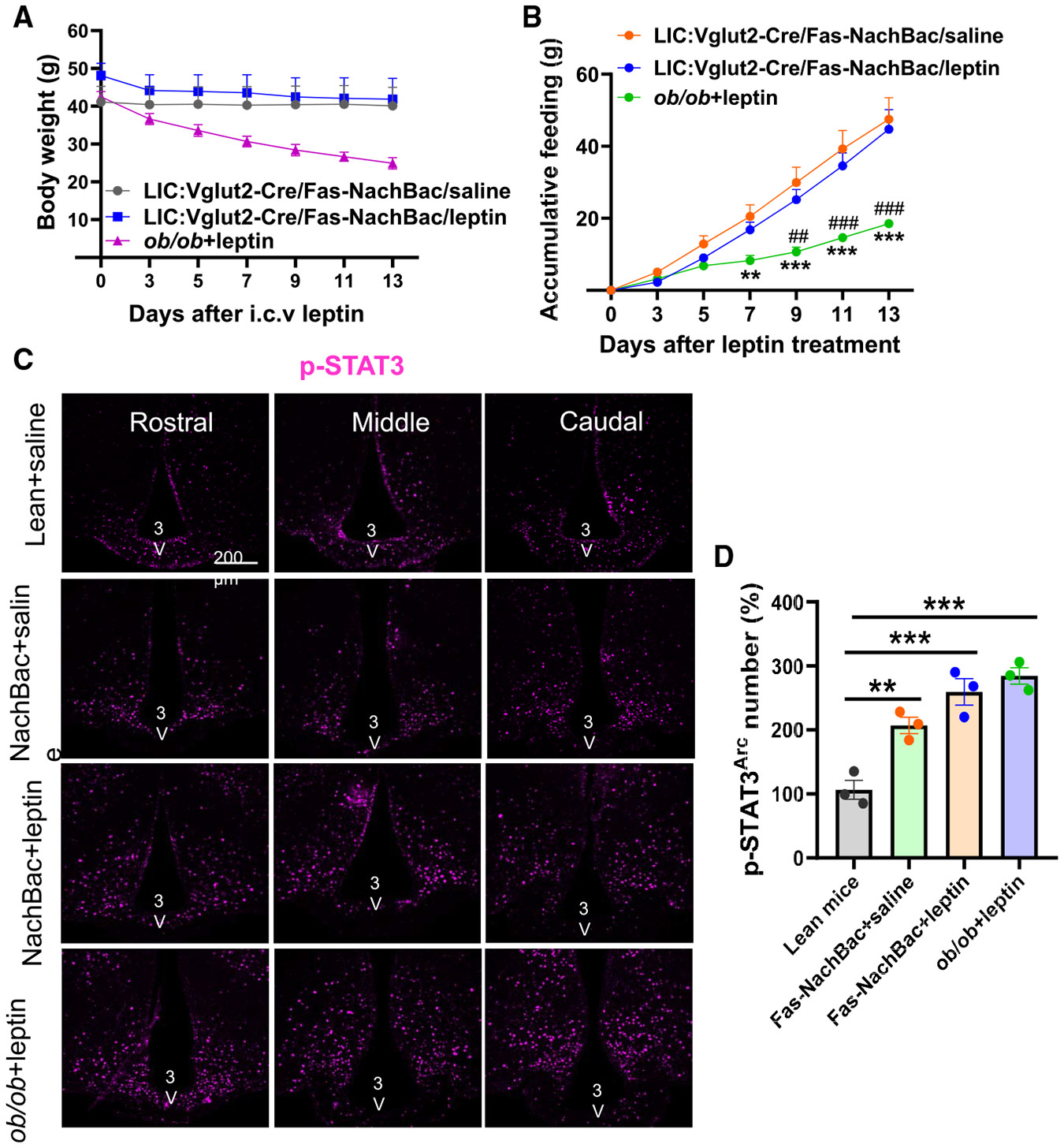
Obesity by activation of Arc non-LepR neurons associated with phenotypic leptin resistance but normal p-STAT3 signaling (A) Changes in body weight of male LIC::Vglut2-Cre mice 8 weeks after NachBac injection treated with i.c.v. saline or leptin minipump as well as body weight-matched male *ob/ob* mice treated with i.c.v leptin minipump for 2 weeks (*n* = 5 or 4 mice for NachBac injection treated with i.c.v. saline or leptin minipump, 3 mice for ob/ob mice treated with leptin as positive control, two-way RM ANOVA, NachBac injection treated with i.c.v. saline vs. leptin, *p* > 0.05). (B) Accumulated food intake measurements on the indicated days after minipump treatments in the same mice in (A) (*n* = 5 mice for NachBac injection treated with i.c.v. saline and *ob/ob* treated with leptin, 4 for NachBac injection treated with leptin; two-way RM ANOVA, NachBac injection treated with i.c.v. saline vs. leptin, *p* > 0.05). (C) Representative pictures showing p-STAT3 immunostaining in the Arc in control, NachBac-injected LIC::Vglut2-Cre mice treated with saline or leptin and *ob/ob* mice treated with leptin in Arc sections at three 3 different bregma levels. (D) Comparison in the number of p-STAT3 expressing neurons in the four groups of mice as described in (C) (*n* = 3 mice/each, one-way ANOVA, lean mice vs. NachBac+saline, **p = 0.0047; lean mice vs. NachBac+leptin, ***p = 0.0003; lean mice vs. *ob/ob*+leptin, ***p = 0.0001). Scale bar: 50 μM. All data are presented as mean ± SEM.

Because we selectively targeted Arc non-LepR neurons in our obesity model, we reasoned that the function of LepR neurons should remain intact and, therefore, leptin action should be maintained. To examine this contention, we performed intracerebroventricular (i.c.v.) leptin infusion in NachBac-injected LIC::Vglut2-Cre mice with leptin-deficient *ob/ob* mice as a control group. Within 2 weeks of leptin infusion, the body weight of *ob/ob* mice exhibited a dramatic reduction, whereas body weights in NachBac-injected mice were largely unaltered (Figure 3A), suggesting that leptin is ineffective in reducing body weight in the severely obese NachBac-injected mice. Consistent with no responses in body weight, food intake measured within the leptin infusion period showed that leptin had no effects on feeding compared to the saline-treated controls (Figure 3B). These data demonstrate that the obesity in NachBac-injected LIC::Vglut2-Cre mice is associated with severe phenotypic leptin resistance.

Given the observed phenotypic leptin resistance in NachBac-injected LIC::Vglut2-Cre mice, we next examined leptin signaling within the Arc. We included leptin-treated *ob/ob* and saline-treated lean wild-type mice as two additional groups for high and baseline leptin sensitivity conditions, respectively. As expected, leptin-treated *ob/ob* mice exhibited significantly more p-STAT3 signal than saline-treated wild-type mice (Figures 3C and 33D). Interestingly, both saline- and leptin-treated NachBac mice exhibited significantly more p-STAT3 signal compared to saline-treated wild-type mice (Figures 3C and 3D). Notably, leptin-treated NachBac mice showed a p-STAT3 expression level comparable to that of leptin-treated *ob/ob* mice (Figures 3C and 3D), demonstrating that, despite severe obesity development and phenotypic leptin resistance, leptin-induced p-STAT3 signaling remains intact in Arc LepR neurons.

Since obesity induced by chronic activation of non-LepR neurons is leptin resistant, we next explored the possibility that non-LepR neurons function directly downstream of LepR neurons. Toward this, we injected a mixture of pAAV-FlpOn/CreOff-EYFP and pAAV-Con/Fon-mCherry viruses into the Arc of Vgat-Flp::LIC mice to label Arc^GABA^ non-LepR and LepR neurons, respectively. These mice were then divided in fed, fasting, and fasting with leptin treatment groups. As expected, fasting caused a significant increase in c-Fos expression in LepR neurons, which were reduced by leptin treatment (Figures S7A-S7C). In contrast, non-LepR neurons were not responsive to fasting or leptin treatment (Figures S7A, S7B, and S7D), suggesting that non-LepR neurons function distinctly from LepR ones in response to fasting. To more directly examine whether non-LepR neurons are a direct downstream target of LepR neurons, we injected a mixture of pAAV-nEF-Con/Fon-ChRmine-oScarlet and pAAV-EF1a-FlpOn/CreOff-GCaMP6f viruses into the Arc of Vgat-Flp::LIC mice to achieve specific expression of ChRmine in LepR neurons and GCaMP6f in non-LepR neurons for fiberphotometry recordings (Figure S8A). With implanted fiberoptics, opto-stimulation of LepR neurons failed to cause Ca^2+^ changes in non-LepR neurons (Figures S8B and S8C). These results collectively suggest that non-LepR neurons are not a direct target of, but rather function in parallel to, LepR neurons in body weight regulation. Consistently, these two subsets of neurons send parallel projections to common downstream sites (Figure S9).

### Chronic inhibition of Arc non-LepR neurons reduced DIO

**Figure 4.**
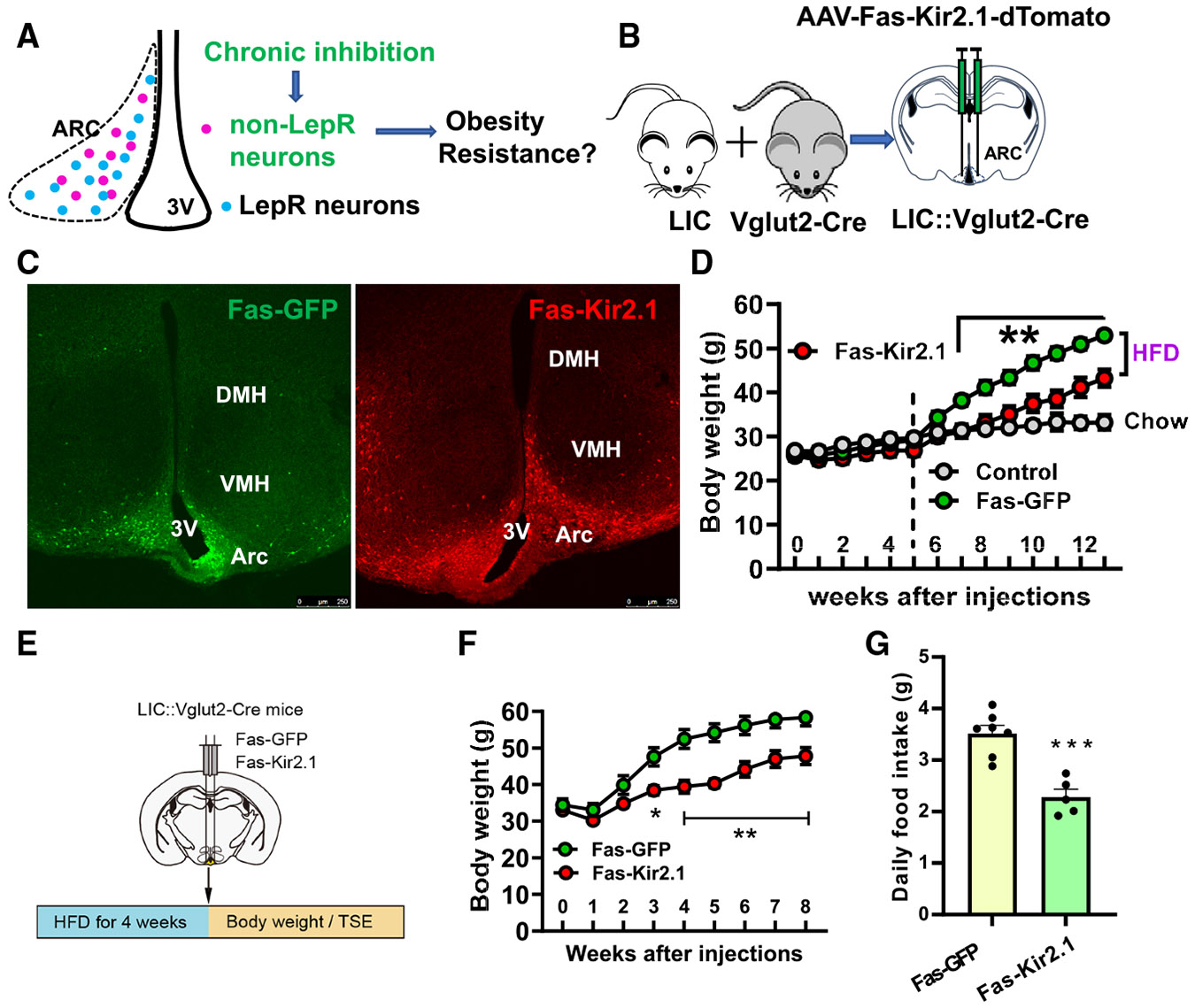
Chronic inhibition of Arc non-LepR neurons caused obesity reduction and reversal by HFD (A) Diagram depicting the experimental design to examine the impact on DIO with specific chronic inhibition of Arc non-LepR neurons. (B) Diagram showing the scheme of chronic inhibition of Arc non-LepR neurons by delivering the Kir2.1 virus to the Arc of LIC::Vglut2-Cre mice. (C) Representative pictures showing the expression of Kir2.1 viral expression in the Arc of control virus (left) and Kir2.1 virus expression (right). (D) Weekly body weight between the injected male mouse groups after viral delivery and then fed 5 weeks on chow followed by 8 weeks of HFD along with a littermate control constantly fed chow (*n* = 5 for chow, *n* = 10 for Fas-GFP, and *n* = 8 for Fas-Kir2.1; two-way ANOVA, *p* > 0.05 for comparisons between Fas-GFP and Fas-Kir2.1 for all weeks on chow and *p* < 0.01 for the comparisons starting from the second week on HFD). (E) Diagram showing the scheme of chronic inhibition of Arc non-LepR neurons with Kir2.1 viral injection after obesity development by HFD for 4 weeks. (F) Weekly body weight between the injected male mouse groups during the 8-week period of continuous HFD feeding (*n* = 7 for Fas-GFP and *n* = 5 for Fas-Kir2.1, one-way ANOVA tests, *p* < 0.05 from the fourth week on HFD). (G) Daily feeding between the two groups of mice (*n* = 5 for Fas-GFP and *n* = 5 for Fas-Kir2.1, unpaired t tests, *p* = 0.00017). All data are presented as mean ± SEM. Scale bar: 50 μM.

Given the HFD-induced activation of Arc non-LepR neurons, we next examined whether reversal of this activation (i.e., inhibition of these neurons) reduces DIO (Figure 4A). Thus, we delivered to the Arc of LIC::Vglut2-Cre mice the pAAV-Syn-Fas-Myc/Kir.21*(E224G,Y242F):P2A: dTomato virus, which expresses Kir2.1 and dTomato in non-Cre neurons (Figure 4B). As expected, the viral expression was largely limited to the Arc for both control and Kir2.1 groups (Figure 4C). To examine the impact of Kir2.1-mediated chronic inhibition on obesity prevention, we delivered the virus first, and then mice were fed chow for 5 weeks, followed by an additional 8 weeks of an HFD (Figure 4D). Kir2.1 expression had no impact on body weight on the chow diet but effectively reduced obesity development on the HFD, albeit the reduction was not in totality (Figure 4D).

To examine the impact on obesity reversal, we induced DIO in LIC::Vglut2-Cre mice by feeding them 4 weeks of an HFD before delivering the Kir2.1 virus (Figure 4E). We then followed the body weight for an additional 8 weeks on an HFD and found that the Kir2.1 mice showed significantly reduced obesity development compared to the control group (Figure 4F), suggesting that chronic inhibition of Arc non-LepR neurons is sufficient to reverse DIO. To understand the mechanism of action in obesity reversal, we subjected the mice to metabolic measurements at a time before body weight difference between the two groups reached significance and found that the Kir2.1 mice showed reduced food intake (Figure 4G) but no significant difference in either O_2_ consumption (Figures S10A and S10B) or locomotion (Figures S10C and S10D). Thus, chronic inhibition of Arc non-LepR neurons is sufficient to reduce DIO.

Given the known diversity of Arc neurons in body weight regulation,^18,25-27^ we next explored the potential neuron subsets that contribute to the observed effect. It has been suggested that a subset of AgRP neurons are LepR negative.^28^ To directly test this, we used NPY-GFP mice to label AgRP neurons and LIC::Ai9 reporter mice to label LepR neurons. In NPY-GFP::LIC::Ai9 reporter mice, we observed a subset of AgRP neurons that was LepR negative (Figure S11), suggesting a potential role of this subset in promoting obesity. Arc tyrosine hydroxylase (TH)-expressing neurons are known to be GABAergic and regulate body weight.^29^ Consistent with previous results,^30^ these neurons were found to be largely LepR negative, and importantly, they were not responsive to the HFD with c-Fos (Figures S12A and S12B). When Arc TH neurons were targeted with NachBac expression in TH-Cre mice, it failed to cause body weight changes (Figures S12C-S12D), suggesting that Arc TH neurons do not contribute to DIO.

## DISCUSSION

Leptin resistance has been suggested to be the major culprit for obesity development in both obese patients and DIO in rodents.^6,16,31^ In this context, it is generally assumed that strategies to reverse leptin resistance will be able to reduce obesity.^6,16^ Thus, extensive efforts have been devoted to understanding the underlying mechanism responsible for leptin resistance, which remains elusive. Our current results suggest that HFD feeding induced activation of Arc non-LepR neurons and that activation of these non-LepR neurons, in turn, caused massive obesity. Parallel studies indicated that inhibition of these neurons effectively prevented and reversed DIO. Of note, the observed acute HFD-induced activation of Arc neurons is consistent with previous reports of augmented activity of both AgRP and prepronociceptin (PNOC) neurons following palatable food exposure.^32,33^ Our results demonstrate that Arc non-LepR neurons contribute significantly to DIO and serve as an alternative neural basis for leptin resistance observed in DIO. Of note, previous studies have suggested the existence of non-homeostatic reward-related hyperphagia and associated obesity development,^34^ which may also serve as an additional neural basis of leptin-insensitive leptin resistance.

LepR neurons are mainly located in the hypothalamus, especially in the Arc, but also found in other brain regions, and therefore, leptin action is thought to be mediated by a distributed brain network.^35^ Supporting this, deletion of LepR in various brain regions causes obesity.^36^ Despite this, in the brain, leptin action is primarily mediated by GABAergic neurons.^37^ Among all brain regions expressing LepR neurons, the Arc represents a key region containing GABAergic neurons.^37^ Indeed, deletion of LepR within the mediobasal hypothalamus causes massive obesity,^38^ reminiscent of LepR-deficient *db/db* mice, suggesting a major role of Arc^GABA^ neurons in mediating the leptin action on body weight. Consistently, the Arc is the major region in the brain that exhibits leptin resistance at the p-STAT3 signaling level.^39-41^ In addition, chronic inhibition of Arc^GABA^ neurons reduces body weight and rescues obesity development by leptin deficiency to a normal level.^18^ Here, we used a combination of mouse genetics, conditional viral vectors, and site-specific virus delivery to achieve specific targeting of Arc neurons; however, it is inevitable that viral vectors may infect nearby areas, including the dorsal medial hypothalamus and tuberal region, in which GABAergic neurons are known to regulate feeding.^42,43^ Thus, a potential contribution from these nearby neurons cannot be completely ruled out. Nonetheless, our observations collectively suggest that Arc^GABA^ neurons represent a key region underlying DIO and leptin resistance.

Previous results suggest that activation of random subsets of Arc^GABA^ neurons causes massive obesity.^18^ Given the major role of Arc^GABA^ neurons in mediating leptin action on body weight regulation and the current results showing a profound role of Arc^GABA^ non-LepR neurons in obesity development, Arc LepR and non-LepR neurons represent two subsets of Arc^GABA^ neurons important for body weight regulation. The non-LepR subset may play a role similar to the known leptin-sensitive AgRP neurons, which, once activated, cause massive obesity.^18,25^ Related to this, *ob/ob* or *db/db* mice, both with functional leptin deficiency, associated with increased neuron activity in the Arc,^22^ presumably due to loss of inhibitory action by leptin on LepR neurons, are severely obese. In addition, our data suggest that non-LepR neurons are not a direct downstream target of and do not respond to LepR neuron activation. Thus, our results support a model in which Arc LepR and non-LepR neurons represent two parallel pathways in mediating body weight, and activation of either is sufficient to cause obesity. In case of *ob/ob* mice with selective loss of leptin action and, hence, activation of Arc LepR neurons, leptin treatment is known to completely correct the obesity phenotype to a normal level, suggesting an effective role of leptin in reducing the aberrant activation of Arc LepR neurons owing to loss of leptin action. In the case of DIO, as our data suggest that HFD feeding increased the activity of Arc non-LepR neurons, leptin, although at an increased level, may not be able to exert a direct inhibitory action on non-LepR neurons and therefore fails to reduce obesity in DIO, causing phenotypic leptin resistance. Thus, our current results reveal a previously unappreciated neural basis underlying leptin resistance.

Our results demonstrate a dissociation between phenotypic leptin resistance and p-STAT3-based leptin sensitivity, raising an important point regarding whether p-STAT3 signaling can be effectively used as a surrogate for leptin resistance. Our results showing that chronic inhibition of Arc^GABA^ non-LepR neurons effectively reduces DIO suggest that leptin, despite the known existence of reduced p-STAT3 signaling, may still be able to reduce the activity of Arc LepR neurons, which otherwise would be sufficient to render obesity, even with inhibition of Arc^GABA^ non-LepR neurons. Supporting this, previous results suggest that the leptin-inhibitory action is independent of pSTAT3 signaling^22^ and, importantly, that antagonism of leptin action effectively increases feeding and obesity in DIO mice, arguing for intact leptin action in DIO states.^44,45^ Interestingly, one previous study suggests that HFD feeding negates the response of *ob/ob* mice to leptin treatment in reducing body weight, which is associated with an intact p-STAT3 response,^46^ suggesting the existence of additional neuron groups in mediating leptin resistance. It will be of interest to examine whether non-LepR neurons fulfill the role. In total, our results reveal Arc^GABA^ non-LepR neurons as an alternative neural basis for leptin resistance and effective therapeutic target against obesity and suggest that improving cellular leptin resistance alone may not be able to reduce DIO.

### Limitations of the study

Despite a conceptual advance in revealing aberrant activation of Arc^GABA^ non-LepR neurons as a potential alternative neural basis underlying leptin resistance, several limitations can be identified in this study. First, the identity of non-LepR neurons that mediate the observed effect remains unknown. Increasing evidence suggests a diverse groups of GABAergic neurons in the Arc that play distinct roles in feeding and body weight regulation,^18,25-27^ and identification of this functional group will provide key insights for understanding the mechanisms underlying DIO toward effective obesity reversal. Of note, our results ruled out a potential contribution from TH neurons. Second, the identity of neurotransmitters that mediate the observed effect also remains to be tested. Since these neurons are all GABAergic, the role of GABA, among others, can be tested. Third, the nature of non-LepR neuron activation by HFD remains unclear. Our data on chronic inhibition effectively leading to obesity reversal suggest sustained activation of these neurons by HFD feeding, which, however, requires further confirmation. It is also unknown whether the activation is in response to the amount of energy intake or the type of diets; i.e., chow vs. HFD. Answers to these questions will further unravel the neural basis for DIO and leptin resistance.

## STAR★METHODS

### EXPERIMENTAL MODEL AND STUDY PARTICIPANT DETAILS

#### Animal care and strains

Mice were housed under a 12 h light/dark cycle at temperature-controlled room (21°C–22°C) and allowed to free access food and water. All animal research followed relevant ethical regulations. Animal care and procedures were approved by Animal Welfare Committe of The University of Texas Health Science center at Houston. Vgat-Cre mice, ob/+ mice, LepR-Cre mice were bred to Vglut2-Cre mice, they all obtained from Jac Laboratory. All mice used for stereotaxic injections were at least 8–10 weeks old.

### METHOD DETAILS

#### Stereotaxic injections and viral vectors

Stereotaxic surgeries to deliver viral constructs were performed as previously described (Zhu et al., Nat Metab, 2020). Briefly, mice were anesthetized with a ketamine/xylazine cocktail (100 mg/kg and 10 mg/kg, respectively), and their heads affixed to a stereotaxic apparatus. Viral vectors were delivered through a 0.5 μL syringe (Neuros Model 7000.5 KH, point style 3; Hamilton, Reno, NV, USA) mounted on a motorized stereotaxic injector (Quintessential Stereotaxic Injector; Stoelting, Wood Dale, IL, USA) at a rate of 30 nL/min. Viral preparations were made by the Baylor NeuroConnectivity Core with sterotype DJ and titered more than ~ 10^12^ particles/mL. Viral delivery was targeted to Arc through four local injections with two each side (200 nL/side, anteroposterior (AP): −1.4 and −1.6 mm, mediolateral (ML): ±0.2 mm, and dorsoventral (DV): −5.9 mm). AAV-EF1a-Flex-Kir2.1-P2A-dTomato was delivered bilaterally into Vgat-Cre mice, AAV-DIO-mCherry as a control virus; pAAV-SYN1>Fas-EGFP:P2A:mNachBac, pAAV-SYN1>Fas-Myc/Kir.21*(E224G Y242F):P2A:dTomato were delivered bilaterally or unilaterally into Arc of LepR-Cre:Vglut2-Cre mice, AAV-Fas-GFP injections as a control group.

#### Body weight studies

Weekly body weight was monitored on all mice fed standard mouse chow (Teklad F6 Rodent Diet 8664, 4.05 kcal/g, 3.3 kcal/g metabolizable energy, 12.5% kcal from fat, Harlan Teklad, Madison, WI) for 8 weeks after viral delivery. For prevention experiment, the mice were fed chow diet for 5 weeks and switched to high-fat diet (HFD, 60% of calories derived from fat, Research Diets, New Brunswick, NJ, D12492) for 7 weeks. Body composition (fat mass and lean mass) was measured at indicated times by using the Echo-MRI system (Echo MRI, Houston, Texas). For treatment experiment, mice first fed with HFD for 2 months induction, the body weight were monitored every week.

#### Food intake measurements

Mice were individually housed after weaning, and daily food intake was monitored for 1 week 3 days after viral delivery. Body weights of these mice were also recorded at the beginning and end of the measurement period. Daily food intakes were calculated as the mean values of the one week measurement. All housing cages were changed daily during the measurement period.

#### CLAMS analysis

Energy expenditure was measured by oxygen consumption by indirect calorimetry. For activation experiment, individual housed mice were maintained on chow diet 3–4 days after viral delivery when there would no significant difference in body weight between groups. Body weight were measured before and after CLAMS measurements. Mice were placed at room temperature (22°C–24°C) in chambers of a Comprehensive Lab Animal Monitoring System (CLAMS, Columbus Instruments, Columbus, OH) with capacity of simultaneous measurement of food intake, O2 consumption and locomotion (beam breaks). Food and water were provided *ad libitum*. Mice were acclimatized in the chambers for at least 24 h prior to data collection. Readings of O2 consumption, locomotion and food intake were ploted and compared between groups.

#### Leptin treatment

For activation experiment, the obese mice with stable body weight changes after Fas-NachBac expression, received implantation of 14-day duration minipums (DURECT Corporation, Cupertino, CA), prefilled with leptin (Dr. Parlow, Harbor-UCLA, CA), which allows slow infusion of leptin (50 ng/h) for 13 days. Mice were measured for feeding and body weight once 2 days. Ob/ob mice were used as positive control of leptin treatment.

#### Immunostaining and imaging

After behavioral experiments were completed, study subjects were anesthetized with aketamine/xylazine cocktail (100 mg/kg and 10 mg/kg, respectively) and transcardially perfused 0.9% normal saline following by 10% formalin. Freshly fixed brains were then harvested and placed in 10% buffered formalin in 4°C overnight for post-fixation, then dehydrated in 30% sucrose solution. Brains were frozen and sectioned into 30 μm slices with a sliding microtome and mounted onto slides for post-hoc visualization of injection sites and cannula placements. Brain sections were immunostained with antibodies against c-Fos (1:2000, #2250S, Cell Signaling Technology, Danvers, MA), pSTAT3 (1:1000, #9145S, Cell Signaling Technology, Danvers, MA). Brain sections with reporter expression and/or immunostained flurosceins were visualized with confocal microscopy (Leica TCS SP5; Leica Microsystems, Wetzlar, Germany). c-Fos and p-STAT3 were counted from 3 matched sections containing rostral, middle and caudal of Arc from individual mice, the counted numbers were averaged and compared between conditions and/or groups.

### QUANTIFICATION AND STATISTICAL ANALYSIS

All data were presented as mean ± SEM. Comparisons were performed using Student’s t tests or 1-way or two-way ANOVA followed by Tukey’s multiple comparison post-hoc tests by using GraphPad Prism.9 (GraphPad Software, La Jolla, CA). Details on number of animals used and statistical methods as well as results are provided in figure legends. *p* < 0.05 was considered significant in all cases.

## Supplementary Material

1

2

## Figures and Tables

**Table:** KEY RESOURCES TABLE

REAGENT or RESOURCE	SOURCE	IDENTIFIER
Antibodies		
Rabbit anti-*c*-Fos	Cell Signaling Technology	#2250s; RRID: AB_2247211
Rabbit anti-pSTAT3	Cell Signaling Technology	#9145S
Bacterial and virus strains		
AAV-EF1a-Flex-Kir2.1-P2A-dTomato	This study	N/A
AAV-DIO-mCherry	Addgene	Plasmid #50459
pAAV-SYN1>Fas-EGFP:P2A:mNachBac	This study	N/A
pAAV-SYN1>Fas-Myc/Kir.21*(E224G Y242F):P2A:dTomato	This study	N/A
AAV-Fas-GFP	This study	N/A
AAV-FlpOn/Cre-Off-EYFP	UNC viral core	AV6217
AAV-Con/Fon-mCherry	Addgene	Plasmid #137132
AAV-nEF-Con/Fon-ChRimine-oScarlet	Addgene	Plasmid #137159
AAV-EF1a-FlpOn/CreOff-GcaMP6f	Addgene	Plasmid #137124
Chemicals, peptides, and recombinant proteins		
Formalin	Fisher	#SF100-4
Leptin	Sigma-Aldrich	L3772
Experimental models: Organisms/strains		
Mouse: Vglut2-Cre	The Jackson Laboratory	RRID: IMSR_JAX:016963
Mouse: Vgat-Cre	The Jackson Laboratory	RRID:IMSR_JAX:016962
Mouse: LepR-Cre	The Jackson Laboratory	RRID:IMSR_JAX:032457
Mouse: ob/+	The Jackson Laboratory	RRID:IMSR_JAX:000632
Mouse: (ROSA)26Sortm9(CAG-tdTomato) reporter	The Jackson Laboratory	RRID:IMSR_JAX:007909
Mouse: NPY-GFP	The Jackson Laboratory	RRID:IMSR_JAX:006417
Mouse: TH-Cre	The Jackson Laboratory	RRID:IMSR_JAX:008601
Software and algorithms		
GraphPad Prism (9.5.1)	GraphPad	RRID:SCR_002798; http://www.graphpad.com/
ImageJ 1.54D	Schneider et al.^1^	https://imagej.org

## Highlights

- Hypothalamic arcuate (Arc) GABAergic neurons can be targeted to reduce DIO
- HFD feeding induces activation of arcuate GABAergic neurons
- Activation of Arc non-LepR neurons is sufficient to cause obesity and leptin resistance
- Inhibition of arcuate non-LepR neurons is sufficient to reduce and reverse DIO
