# Supplementary material for: An alternative neural basis underlying leptin resistance
Source: Cell Rep. Author manuscript; Available in PMC 2025 Aug 11. (PMC12337207; doi:10.1016/j.celrep.2025.115863)
Supplement: 1 [file NIHMS2099503-supplement-1.pdf]

Cell Reports, Volume *44*

Supplemental information

An alternative neural basis
underlying leptin resistance

Hongli Li, Cunjin Su, Yuanzhong Xu, Mette Q. Ludwig, Jon Davis, and Qingchun Tong

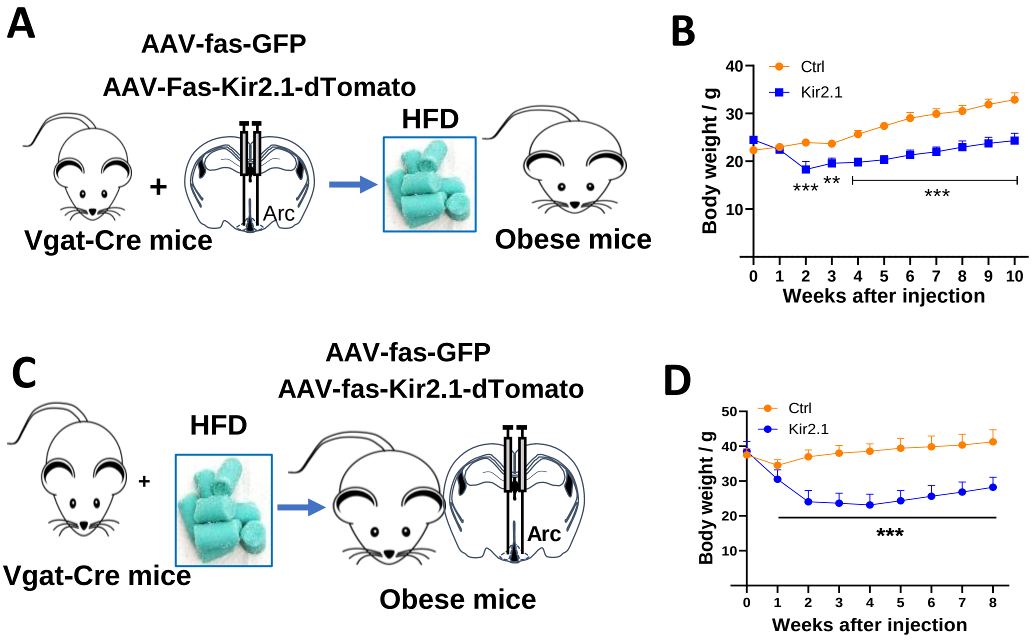

Supplementary Fig. 1. Chronic inhibition of Arc GABAergic neurons caused obesity reduction and reversal on DIO, related to Fig. 1. **A**. Diagram depicting experimental scheme in which AAV-DIO-Kir2.1-dTomato or control viral vectors will be delivered to the Arc of Vgat-Cre mice following by HFD feeding to examine the impact on DIO. **B**. Weekly body weight of the two groups of mice during the 10-week period of HFD feeding (n = 4 mice/each, two-way ANOVA, mCherry vs. Kir2.1, ***$p < 0.001$ at two weeks post viral injection; **$p = 0.001$ at three weeks post viral injection; ***$p < 0.001$ at 4-10 weeks post viral injection). **C**. Diagram depicting experimental scheme in which AAV-DIO-Kir2.1-dTomato or control viral vectors will be delivered to the Arc of DIO Vgat-Cre male mice with 10-week HFD feeding to examine the impact on DIO reversal. **D**. Weekly body weight of the two groups of mice during the 8-week period of HFD feeding (n = 5 mice/each, two-way ANOVA, mCherry vs. Kir2.1, ***$p < 0.001$ at 2-8 weeks post viral injection).

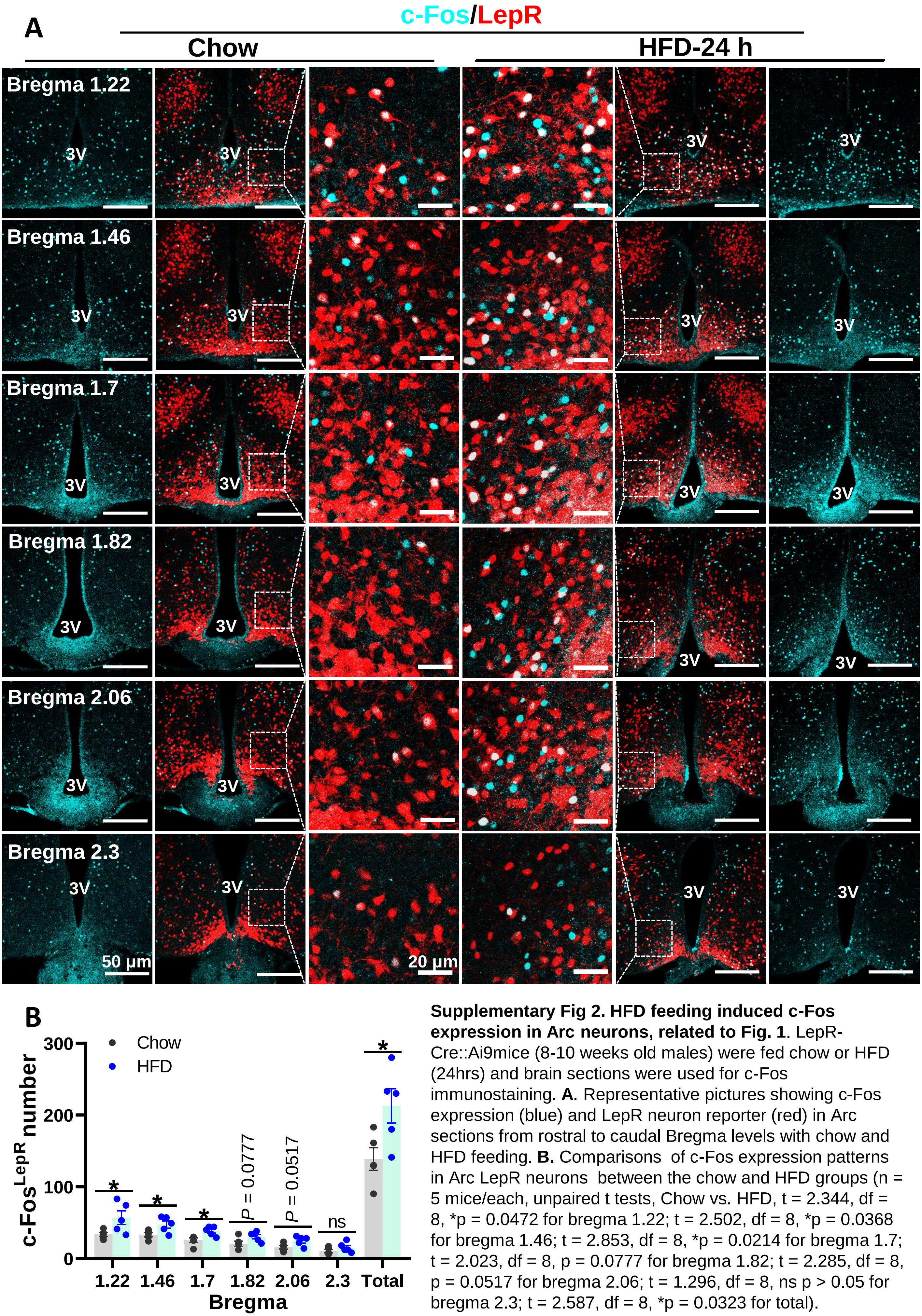

Supplementary Fig 2. HFD feeding induced c-Fos expression in Arc neurons, related to Fig. 1. LepR-Cre::Ai9mice (8-10 weeks old males) were fed chow or HFD (24hrs) and brain sections were used for c-Fos immunostaining. **A.** Representative pictures showing c-Fos expression (blue) and LepR neuron reporter (red) in Arc sections from rostral to caudal Bregma levels with chow and HFD feeding. **B.** Comparisons of c-Fos expression patterns in Arc LepR neurons between the chow and HFD groups (n = 5 mice/each, unpaired t tests, Chow vs. HFD, t = 2.344, df = 8, *p = 0.0472 for bregma 1.22; t = 2.502, df = 8, *p = 0.0368 for bregma 1.46; t = 2.853, df = 8, *p = 0.0214 for bregma 1.7; t = 2.023, df = 8, p = 0.0777 for bregma 1.82; t = 2.285, df = 8, p = 0.0517 for bregma 2.06; t = 1.296, df = 8, ns p > 0.05 for bregma 2.3; t = 2.587, df = 8, *p = 0.0323 for total).

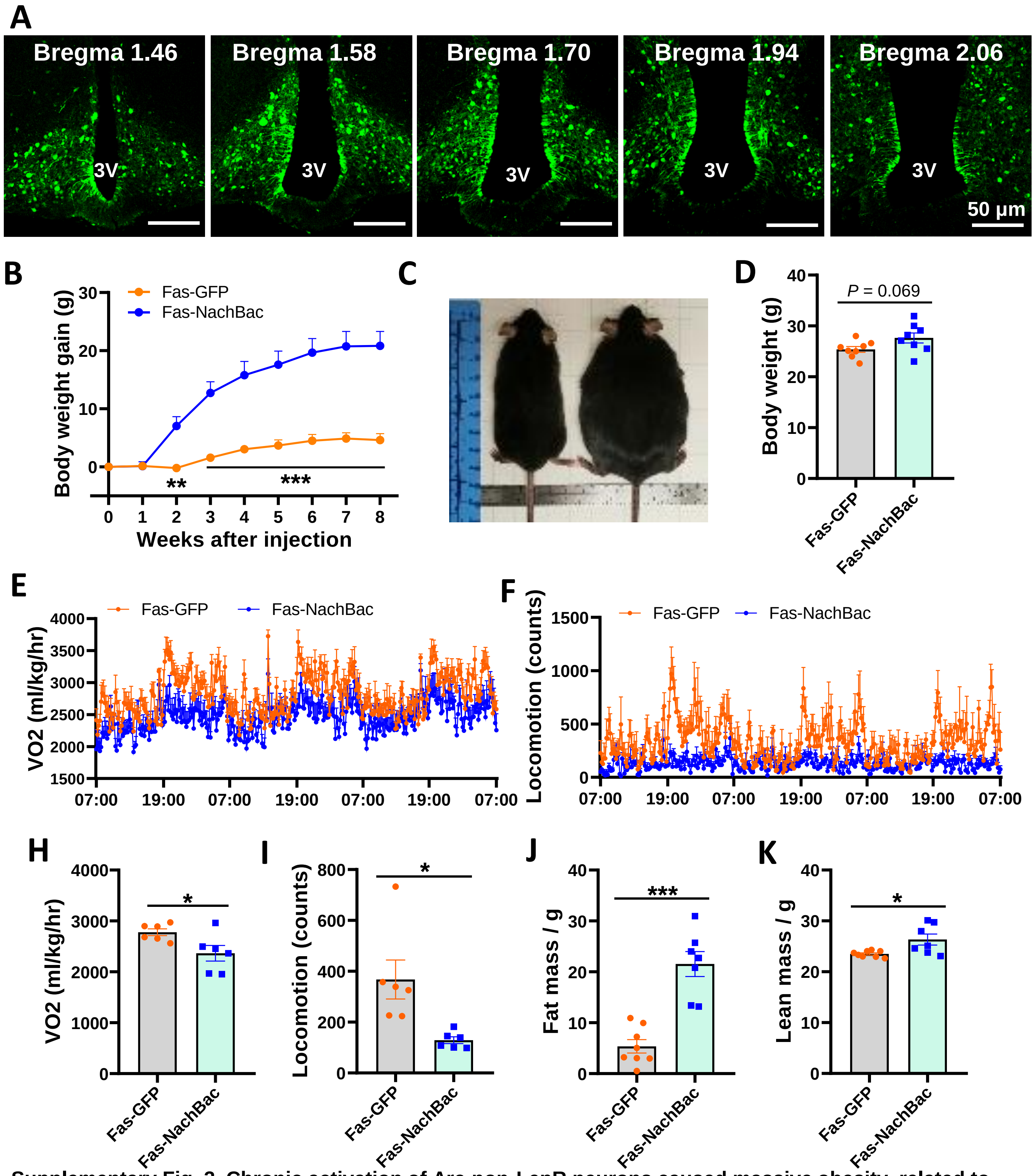

Supplementary Fig. 3. Chronic activation of Arc non-LepR neurons caused massive obesity, related to Fig. 2. A. Representative pictures showing expression patterns of the NachBac viral vectors in the Arc of LepR-Cre::Vglut2-Cre mice from rostral to caudal Bregma levels. **B**. Weekly body weight gains of male LepR-Cre::Vglut2-Cre male mice after viral delivery during the 8-week chow feeding period (n = 8 mice/each, two-way ANOVA, Fas-GFP vs. Fas-NachBac, $**p < 0.009$ at 2 weeks post viral injection; $***p < 0.001$ at 3-8 weeks post viral injection). (**C**) The picture of Fas-GFP and Fas-NachBac-injected mice shown in B at 10 weeks after viral delivery. (**D-I**) Viral injected LepR-Cre::Vglut2-Cre mice were measured in CLAMS (Columbus, Ohio) during the first week after the viral delivery before body weight difference between the groups reached a significance difference (**D**, n = 8/each, unpaired Student's *t* tests, t = 1.969, df = 14, $*p = 0.0691$); real time O2 consumption measurements (**E**) and the associated statistical comparison (**H**, n = 6/each, unpaired Student's *t* tests, t = 2.452, df = 10, $*p = 0.0341$) during the 2-day measurement period; and real time locomotioncounting (**F**) and the associated statistical comparison (**I**, n = 6/each, unpaired Student's *t* tests, t = 3.060, df = 10, $*p = 0.012$). (**J-K**) Comparison in fat mass (**J**, n = 8 for Fas-GFP and 7 for Fas-NachBac, unpaired Student's *t* tests, t = 6.066, df = 13, $***p < 0.001$), lean mass (**K**, n = 8 for Fas-GFP and 7 for Fas- NachBac, unpaired Student's *t* tests, t = 2.725, df = 13, $***p = 0.0173$) in the mice shown in B at 10 weeks after viral delivery.

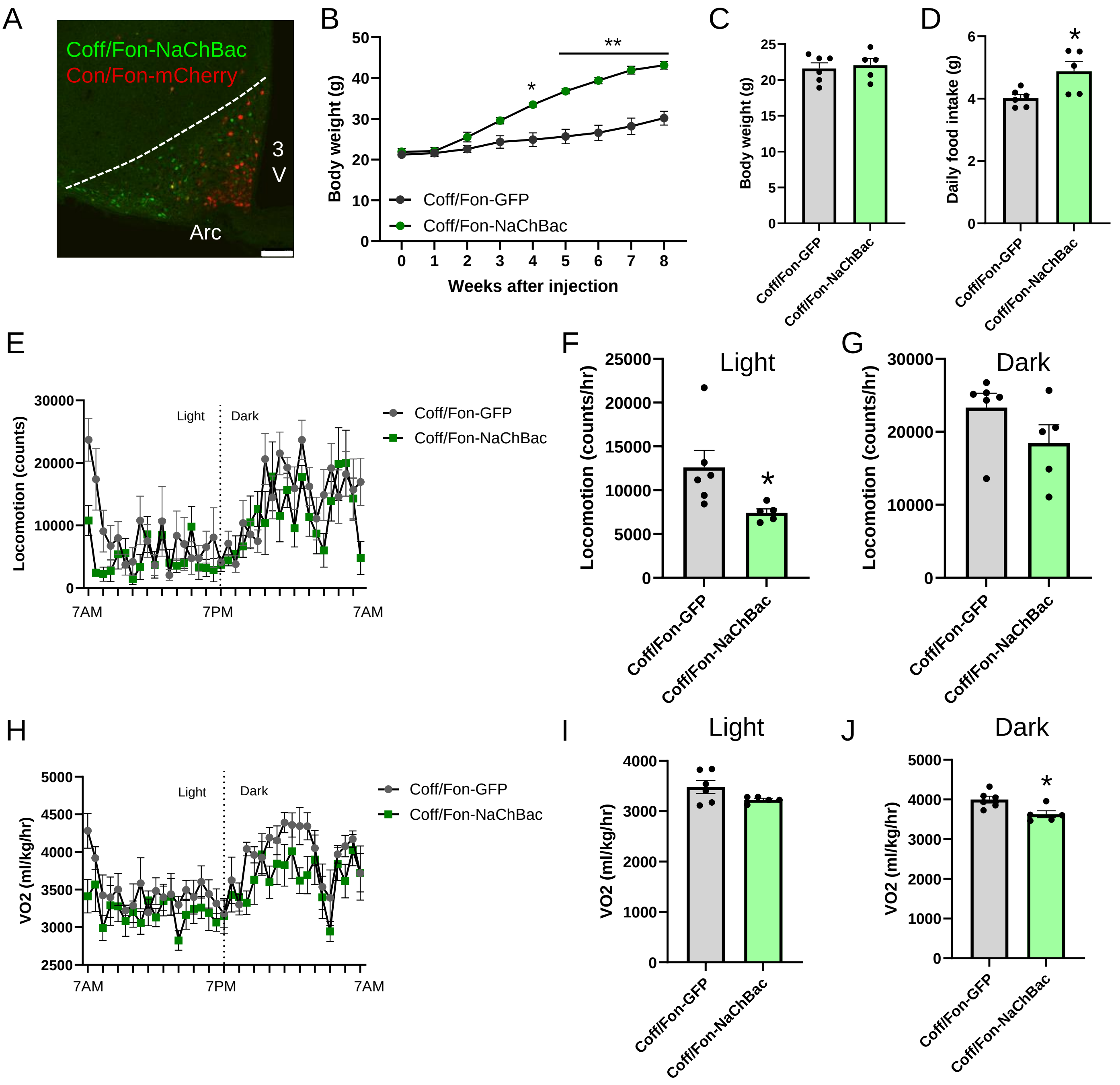

Supplementary Fig. 4. Metabolic cage test after activation of GABAergic non-LepR neurons in the arcuate nucleus, related to Fig. 2. The AAV-CreOff:FlpOn-NaChBac and AAV-CreOn:FlpOn-mCherry virus was delivered into arcuate of Vgat-Flp::LIC (Leptin-Ires-Cre) mice, and the metabolic cage test was performed 2 weeks after injection.

(A) The expression pattern for NaChBac and cFos in the arcuate. Scale bar=100 µm.

(B) Weekly body weight of female mice during 8-week chow feeding period (n=6 mice/GFP group, n=5/NaChBac group, two-way ANOVA, *p=0.023 at 4th week, **p<0.01 at 5-8 weeks post injection).

(C) The initial body weight had no significant difference for metabolic cage test (unpaired t-test, p=0.707).

(D) Activation of GABAergic non-LepR neurons in the arcuate induced daily food intake (unpaired t-test, p=0.021).

(E) Real-time locomotor activity patterns.

(F,G) Comparisons in the locomotor activity measured during light and dark phase. Unpaired t-test, p=0.042 **(F)**.

(H) Real-time O2 consumption patterns.

(I,J) Comparisons in the O2 consumption measured during light and dark phase. Unpaired t-test, p=0.014 **(J)**.

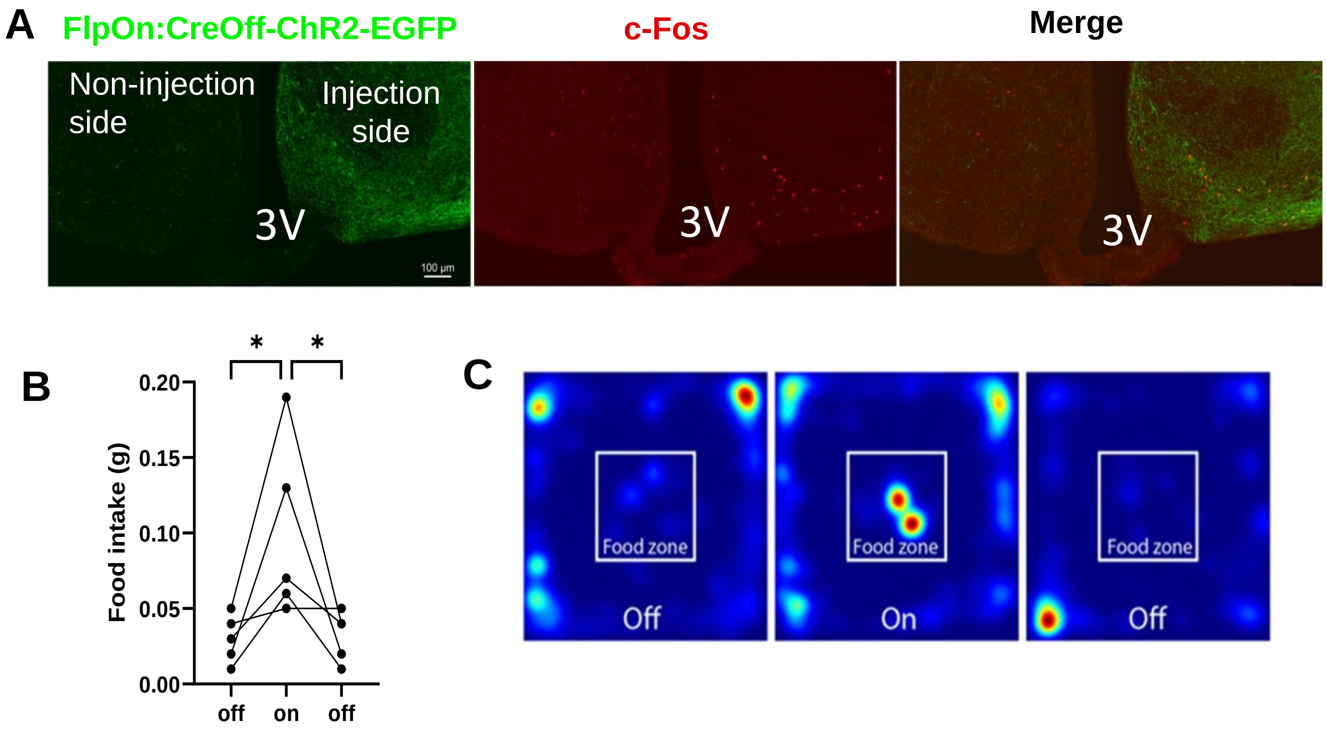

Supplementary Fig 5. In vivo activation of GABAergic non-LepR neurons in the arcuate induced feeding, related to Fig. 2. A) AAV-FlpOn:CreOff-ChR2-EGFP virus was unilaterally delivered into the arcuate nucleus of Vgat-Flp::LIC (Leptin-Ires-Cre) mice. Representative expression of ChR2 and c-Fos after optogenetic stimulation. (B) Comparisons in the amount of food intake between laser off and on (5 Hz, 100 ms, 20 min). N=5, *p<0.05. (C) Representative moving tracks of mice during laser off or on.

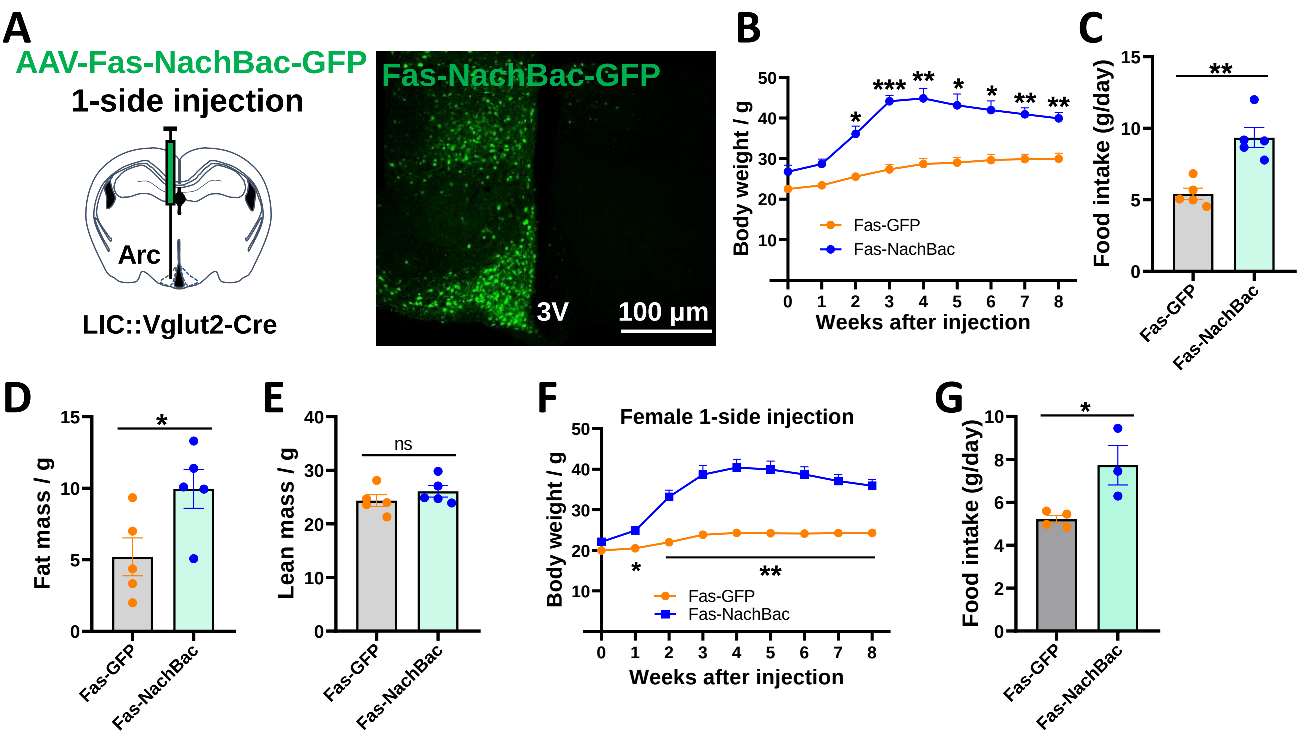

Supplementary Fig. 6. One side activation of Arc non-LepR neurons caused obesity in both males and females, related to Fig. 2. **A**. Diagram showing the scheme of delivering AAV-Fas-NachBac-GFP or control viral vectors to one side of the Arc of 7-8 week old LepR-Cre::Vglut2-Cre mice (left panel) and representative pictures showing NachBac viral expression patterns (right panel). (**B-E**) Phenotypes on comparisons in weekly body weight (**B**, n = 5 mice/each, two-way ANOVA, Fas-GFP vs. Fas-NachBac, p = 0.44 and 0.17 at zero and first week post viral injection, respectively; ***p < 0.001 at 2-8 weeks post viral injection), food intake (**C**, n = 5 mice/each, unpaired Student's t tests, t = 4.840, df = 8, **p = 0.0013), fat mass (**D**, n = 5 mice/each, unpaired Student's t tests, t = 2.508, df = 8, *p = 0.0365) and lean mass (**E**, n = 5 mice/each, unpaired Student's t tests, t = 1.133, df = 8, p = 0.29) in male groups of mice. (**F-G**) Comparisons in body weight (**F**, n = 6 mice/each, two-way ANOVA, Fas-GFP vs. Fas- NachBac, p = 0.93 and 0.17 at zero and first week post viral injection, respectively; ***p < 0.001 at 2-8 weeks post viral injection) and food intake (**G**, n = 4 and 3 mice for Fas-GFP and Fas-NachBac, respectively; unpaired Student's t tests, t = 3.134, df = 5, *p = 0.0258) in female groups of mice. LIC: LepR Cre mice.

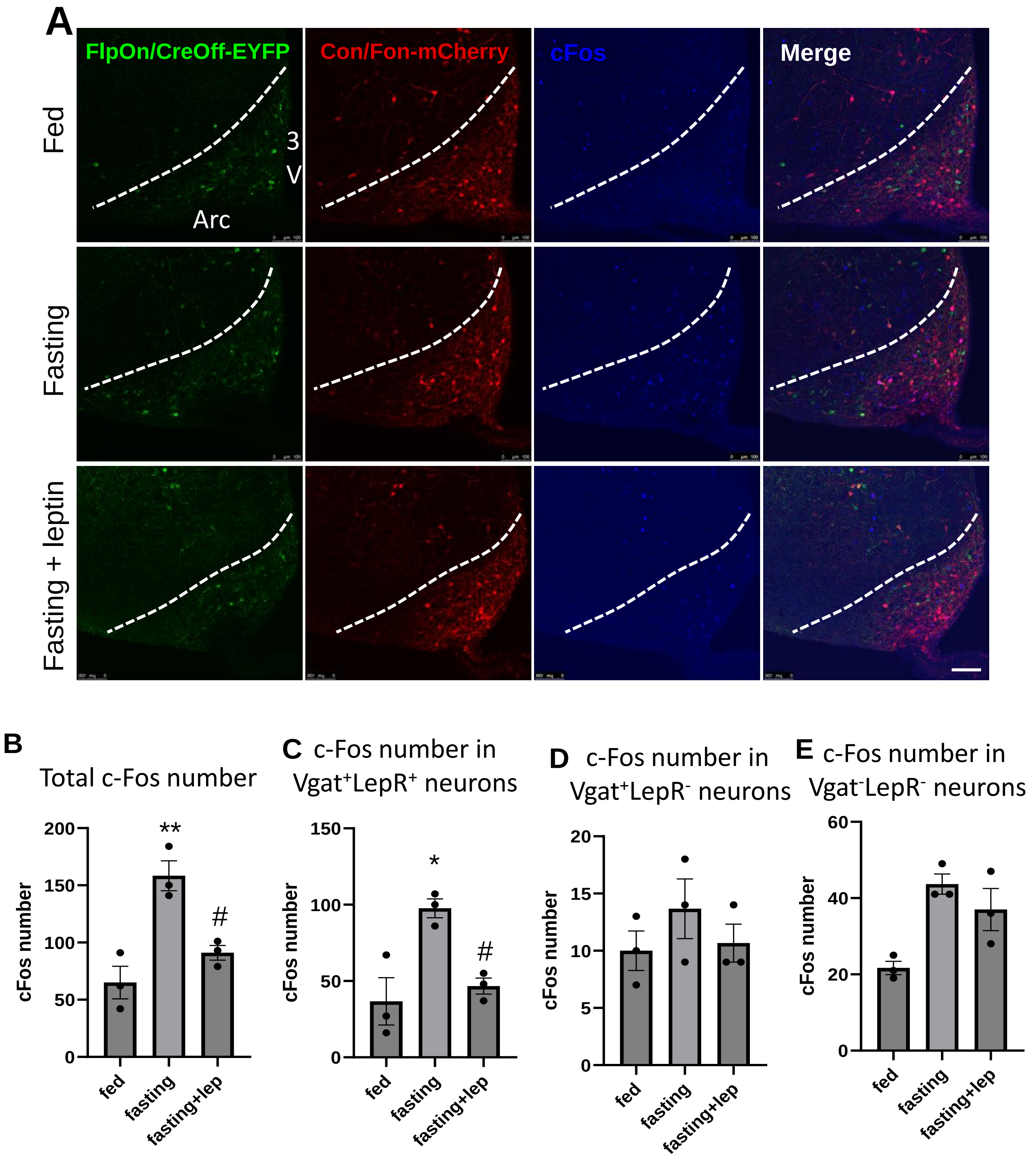

Supplementary Fig. 7: Responses of c-Fos in Arc GABAergic LepR and non-LepR neurons to fasting and leptin treatments, related to Fig. 3. Vgat-Cre::LepR-Ires-Cre (LIC) male mice (8-10 weeks old) were injected with AAV-FlpOn/CreOff-EYFP and AAV-Con/Fon-mCherry to the Arc to label Arc GABAergic non-LepR and LepR neurons, respectively. Three weeks after the injection, these mice were fed ad libitum, fasting, or fasting with leptin treatments, and then perfused for immunostaining for c-Fos. A) Representative pictures showing non-LepR neurons (green), LepR neurons (red), c-Fos expression (blue). B-E) Quantitative comparisons between the 3 groups in total c-Fos number (B), c-Fos number in LepR neurons (C), non-LepR neurons (D), and other neurons (E). 3V: the third ventricle, scale bar= 100 uM, N=3 each, **p<0.01 and *p<0.05 (fed vs fasting), #p<0.05 (fasting vs fasting+leptin), one way ANOVA.

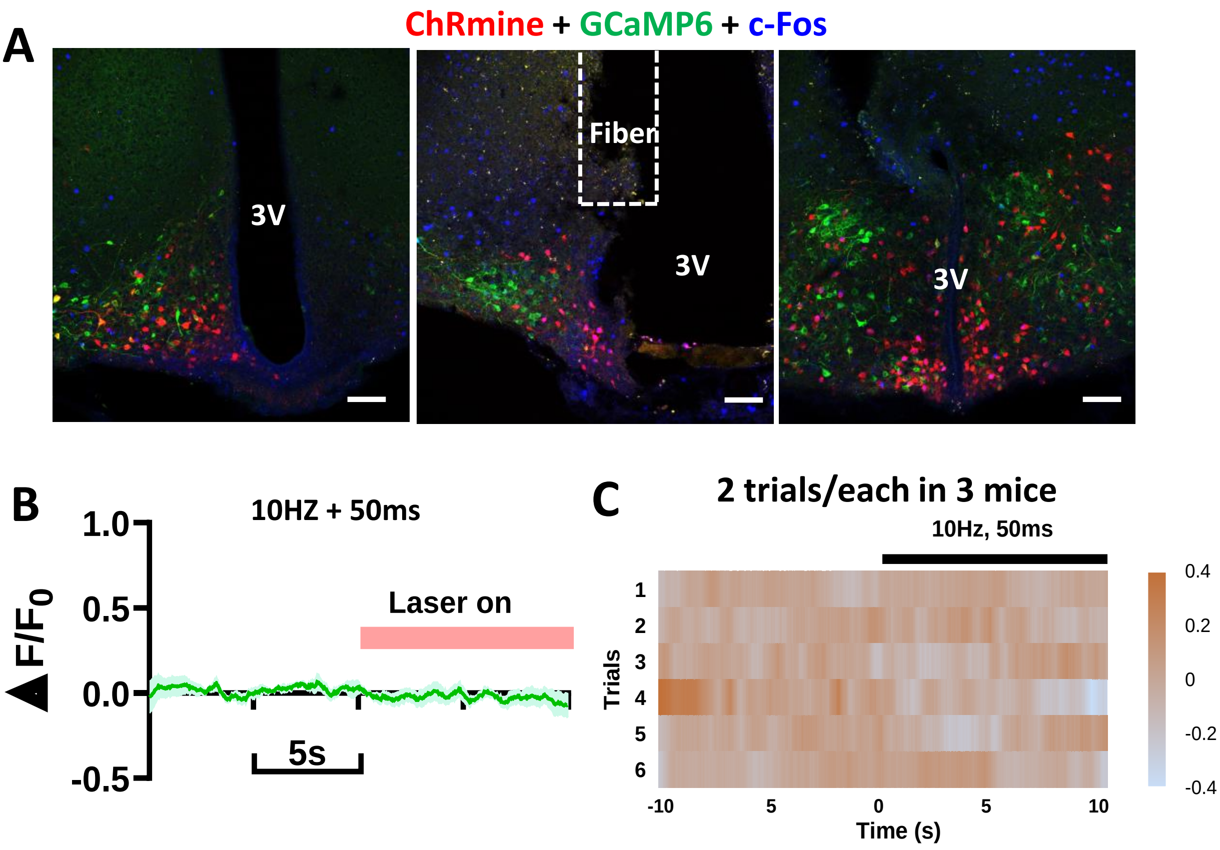

Supplementary Fig. 8: Activity monitoring of Arc GABAergic non-LepR neurons in response to optogenetic activation of Arc GABAergic LepR neurons, related to Fig. 3. Vgat-Flp::LepR-Ires-Cre (LIC) male mice (8-10 weeks old) were injected AAV-nEF-Con/Fon-ChRmine-oScarlet and AAV-EF1a-CreOff/FlpOn-GCaMP6f to the Arc to express ChRmine in GABAergic LepR neurons and GCaMP6f to GABAergic non-LepR neurons, respectively. A) Expression patterns of ChRmine and GCaMP6f in the Arc at respective rostral, middle and caudal levels with c-Fos induction by optostimulation. B) Recording traces of GCaMP6f signal with and without laser stimulation. C) Heat map showing a summary of GCaMP6f signal responses in 3 mice. 3V: the third ventricle; scale bar=100uM.

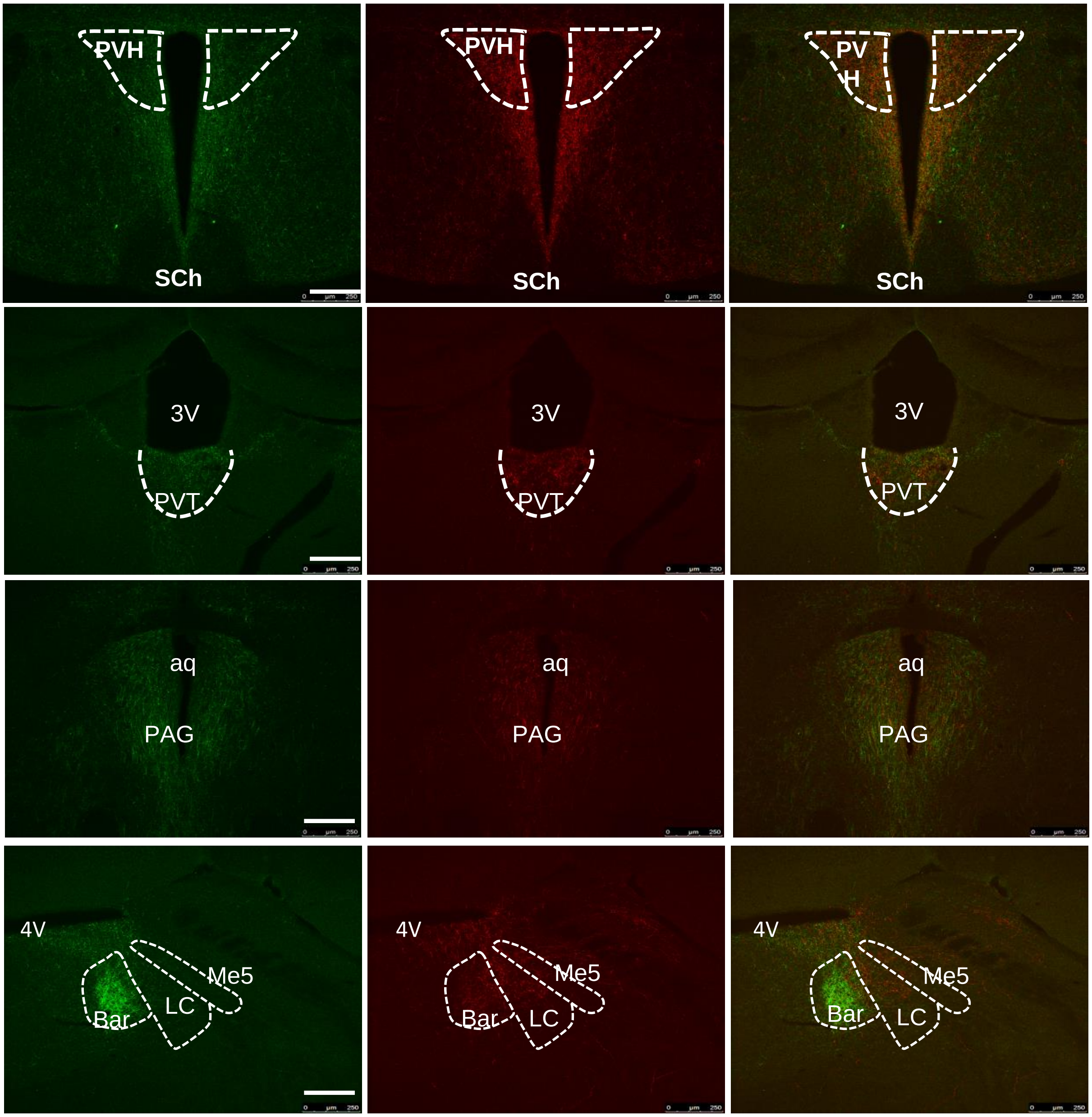

Supplementary Fig. 9: Parallel projections from Arc GABAergic non-LepR neurons and LepR neurons, related to Fig. 3. Vgat-Flp::LepR-Ires-Cre (LIC) male mice (8-10 weeks old) were injected with AAV-Con/Fon-mCherry and AAV-CreOff/FlpOn-GFP to the Arc to express mCherry in GABAergic LepR neurons and GFP to GABAergic non-LepR neurons, respectively. Projections from the 2 subsets of Arc neurons were observed in the PVH, PVT and locus coerulues area. Notably, while most areas show comparable projections, the Bar area receives more projections from non-LepR neurons, compared to LepR ones. PVH: paraventricular hypothalamus; PVT: paraventricular thalams; PAG: periaqueductral grey; SCh: superiorchiasmatic nucleus; LC: locus coeruleus; Me5: mesencephalic trigeminal nucleus; Bar: Barrington's nucleus. 3V: the third ventricle; scale bar=100uM.

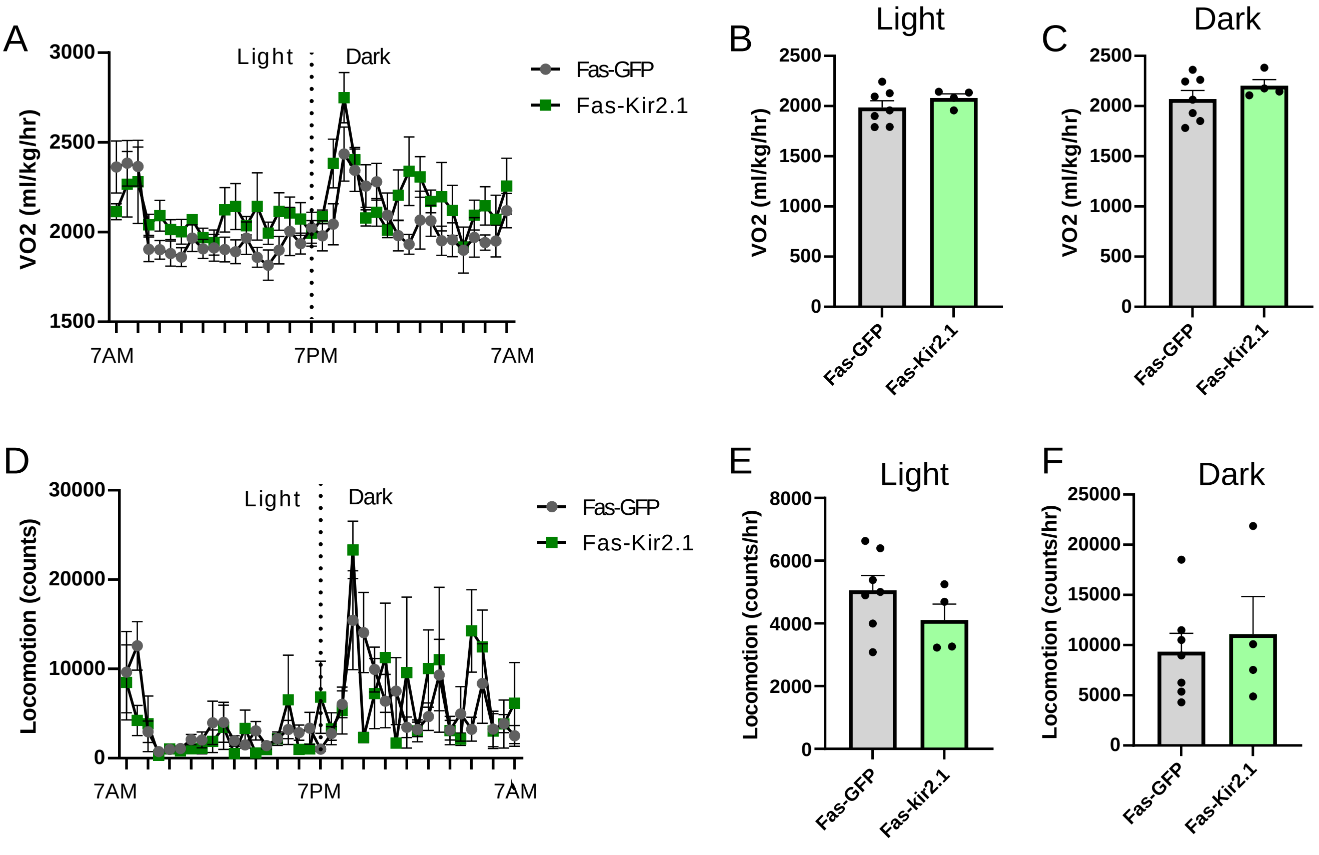

Supplementary Fig. 10: Measurements in TSE phenomaster cages of DIO mice with chronic inhibition of Arc non-LepR neurons, related to Fig. 4. LepR-Ires-Cre::Vglut2-Cre male mice (7-8 weeks old) were first fed HFD for 4 weeks and then injected with AAV-Fas-Kir2.1 or control vectors to the Arc, and then measured in TSE phenomaster cages for O2 consumption and locomotion before body weight difference became significant. (A-C) Real time readings of O2 consumption (A) and the associated statistical comparison (B, C, n = 7 for Fas-GFP and 4 for Fas-Kir2.1, two-way ANOVA, p> 0.05).(D-F) Real time reading of activity counts (D) and the associated statistical comparison (E, F, n = 7 for Fas-GFP and 4 for Fas-Kir2.1, two-way ANOVA, p> 0.05).

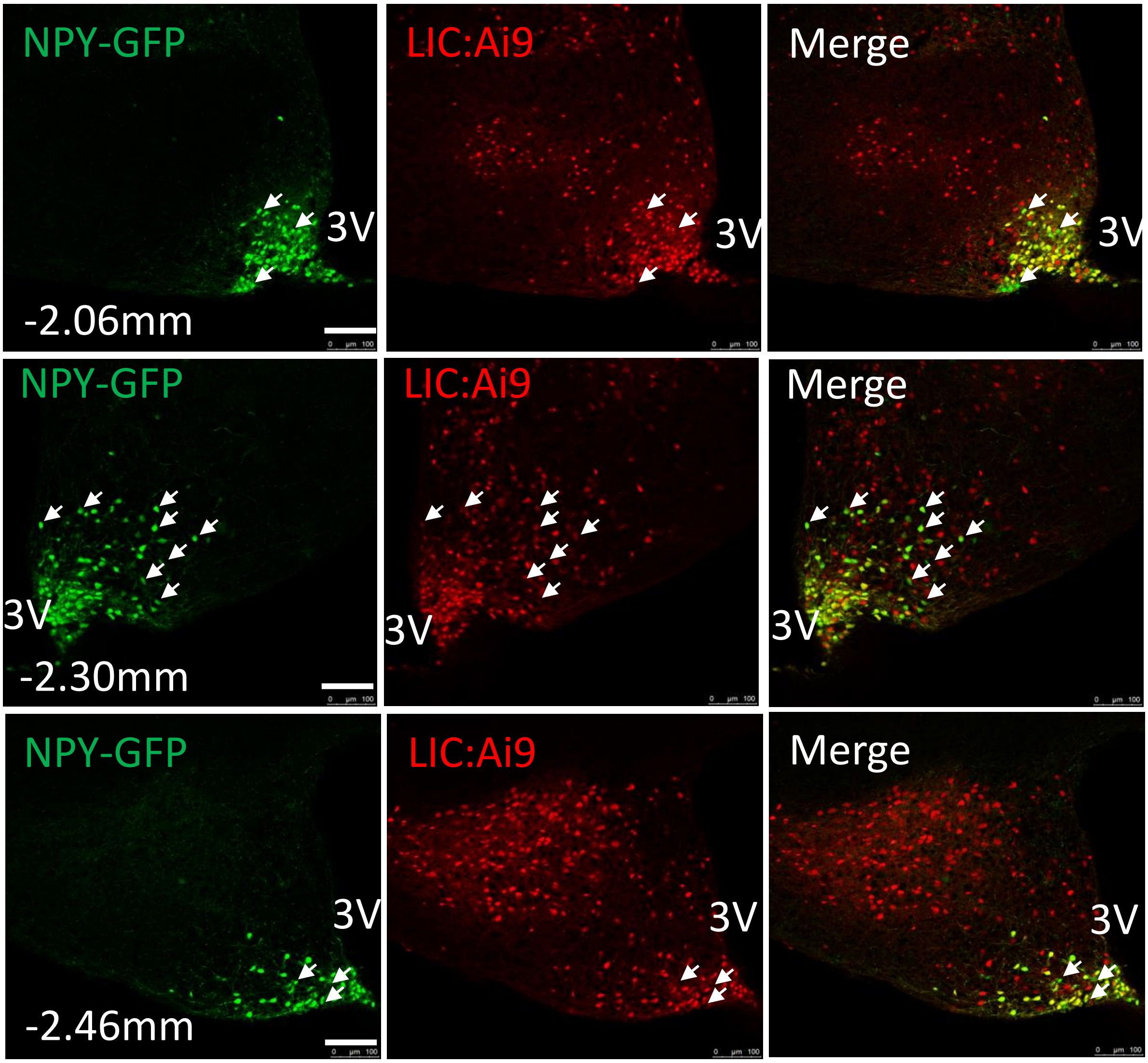

Supplementary Fig. 11: A subset of AgRP neurons represent part of Arc GABAergic non-LepR neurons, related to Fig. 4. NPY-GFP mice were bred with LepR-Ires-Cre (LIC)::Ai9 reporter mice to generate NPY-GFP::LIC::Ai9 mice. Pictures from the 3 indicated bregma levels with GFP (i.e. AgRP/NPY neurons) and Ai9 expression (red, i.e. LepR neurons). White arrows point to GFP positive neurons that are not Ai9 positive. Scale bar= 100 uM, 3V: the third ventricle.

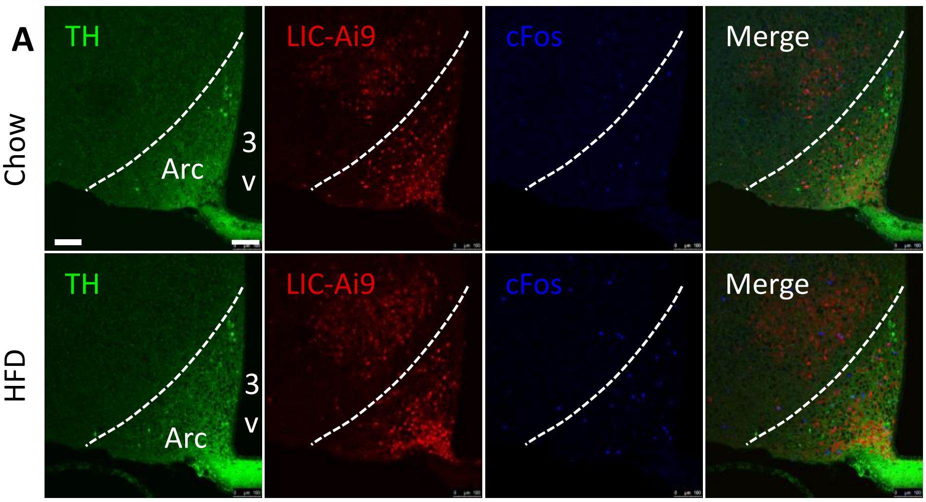

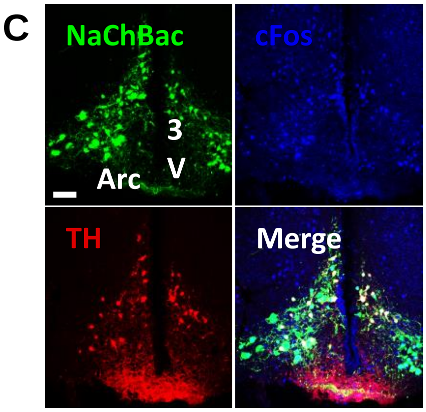

B

	$LepR^+$ ($LepR^+$/$cFos^+$)	TH^+ (TH^+/$cFos^+$)	$LepR^-$/TH^-/$cFos^+$
**Chow**	673.1±62.9 (33.0±5.6)	39.7±1.5 (3.0±1.7)	3.3±1.5
**HFD**	637.3±32.1 (42.3±10.1)	45.8±6.1 (7.0±2.4)	48.3±10.0

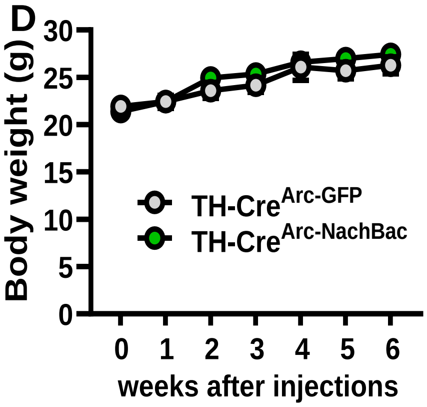

Supplementary Fig. 12. Arcuate tyrosine hydroxylase (TH) expressing neurons represent a subset of Arc GABAergic non-LepR neurons but do not contribute to obesity, related to Fig. 4. A-B) Brain sections from LepR-Ires-Cre::Ai9 mice fed with chow or 24 hr HFD were used for immunostaining for TH (green) and c-Fos (blue) and the presentative pictures were shown in A and the statistical comparison in counting number of neurons with c-Fos, TH and LepR reporter Ai9 (red) were shown in B (of note, due to different conditions for immunostaining and picture taking procedures performed by different experimenters, the absolute number of c-Fos appeared to be less in this experiment compared to that shown in Fig.1; however, the result on increased c-Fos in Arc non-LepR neurons by HFD remains). C-D) Expression of NachBac was confirmed with the associated expression of TH and c-Fos in Arc TH neurons by injections of Cre-dependent NachBac in the Arc of TH-Cre mice (C) and weekly body weight with NachBac expression in TH neurons (D). N=3-4 in A-B and 5-6 in C-D. Scale bar=100 uM, 3V: the third ventricle.
